# Supplementary material for: Immune phenotypes that are associated with subsequent COVID-19 severity inferred from post-recovery samples
Source: Nat Commun. 2022 Nov 25;13:7255. doi: 10.1038/s41467-022-34638-2 (PMC9700777; doi:10.1038/s41467-022-34638-2)
Supplement: Supplementary file 1 — Supplementary Information [file 41467_2022_34638_MOESM1_ESM.pdf]

Immune phenotypes that are associated with subsequent COVID-19 severity inferred from post-recovery samples.

Supplementary Material

Thomas Liechti[1], Yaser Iftikhar[1], Massimo Mangino[2,3], Margaret Beddall[1], Charles W. Goss[4], Jane A. O'Halloran[5], Philip A. Mudd[6,7], Mario Roederer[1]

[1]ImmunoTechnology Section, Vaccine Research Center, NIAID, NIH, USA, 20892
[2]Department of Twin Research & Genetic Epidemiology, King's College of London, London, UK
[3] NIHR Biomedical Research Centre at Guy's and St Thomas' Foundation Trust, London SE1 9RT, UK
[4]Division of Biostatistics, Washington University School of Medicine, St. Louis, MO, USA,
[5]Division of Infectious Diseases, Department of Internal Medicine, Washington University School of Medicine, St. Louis, MO, USA
[6]Department of Emergency Medicine, Washington University School of Medicine, St. Louis, MO, USA, 63110
[7]Center for Vaccines and Immunity to Microbial Pathogens, Washington University School of Medicine, St. Louis, MO, USA, 63110

*E-mail: roederer@nih.gov and liechti.thom@gmail.com

Keywords
SARS-CoV2, COVID-19, Immunophenotyping, Chemokine Receptors, High-dimensional flow cytometry

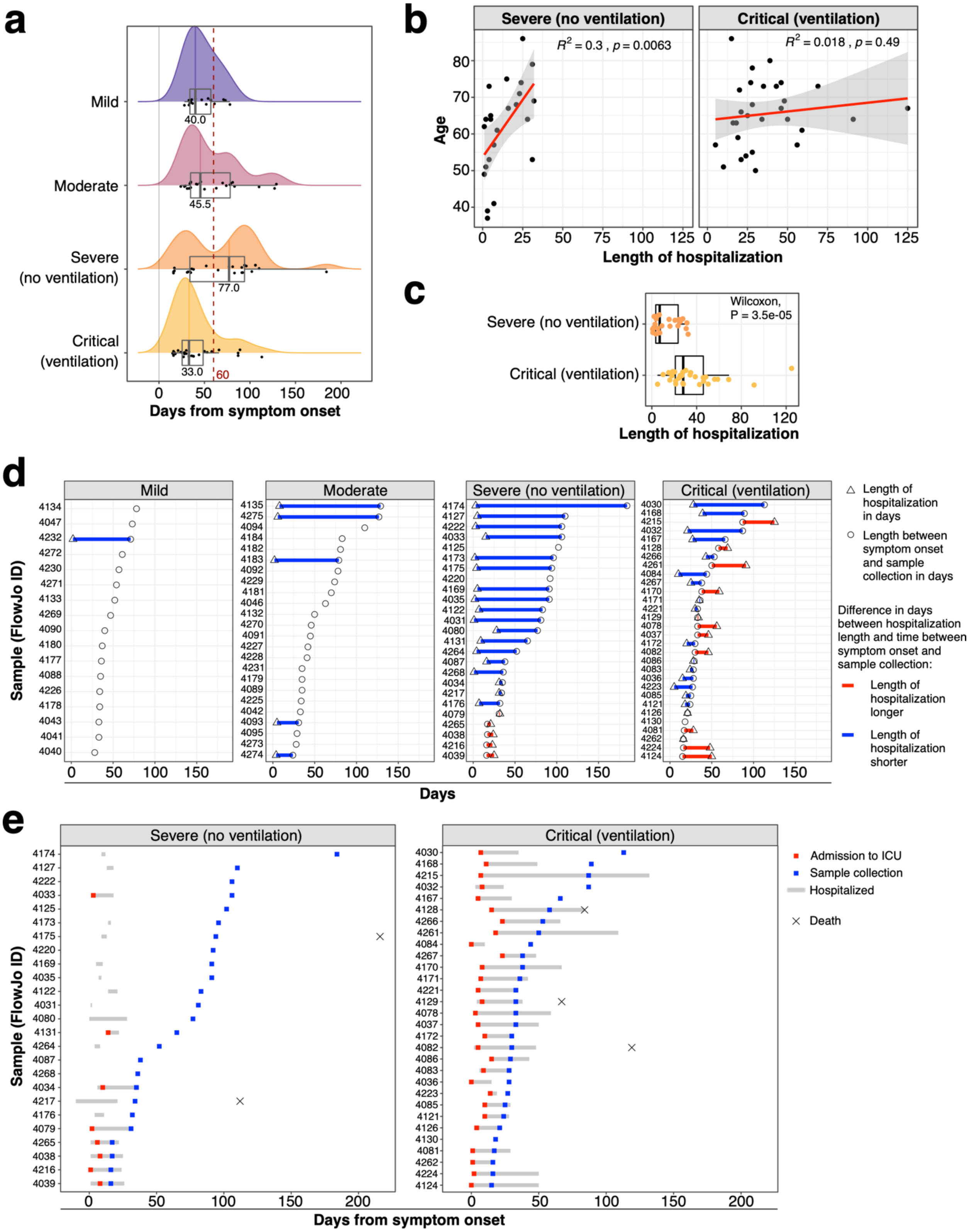
a
Mild
Moderate
Severe
(no ventilation)
Critical
(ventilation)
40.0
45.5
77.0
33.0
60
0
50
100
150
200
Days from symptom onset
b
Severe (no ventilation)
Critical (ventilation)
R^2 = 0.3 , p = 0.0063
R^2 = 0.018 , p = 0.49
Age
Length of hospitalization
c
Severe (no ventilation)
Critical (ventilation)
Wilcoxon,
P = 3.5e-05
Length of hospitalization
d
Mild
Moderate
Severe (no ventilation)
Critical (ventilation)
Sample (FlowJo ID)
Days
Length of hospitalization in days
Length between symptom onset and sample collection in days
Difference in days between hospitalization length and time between symptom onset and sample collection:
Length of hospitalization longer
Length of hospitalization shorter
e
Severe (no ventilation)
Critical (ventilation)
Sample (FlowJo ID)
Days from symptom onset
Admission to ICU
Sample collection
Hospitalized
Death

Supplementary Figure 1: Cohorts and timing of sample collection

a) Distribution of days between symptom onset and sample collection (x-axis) is shown for each COVID-19 severity group (y-axis; mild, moderate, severe and critical COVID-19) as boxplot. Individual datapoints are highlighted as black dots. In addition, histograms (y-axis) depict the distribution of samples for each COVID-19 severity group. The number below each boxplot shows median days between symptom onset and sample collection per group. Red dashed line indicates 60 days cutoff which was used for analysis shown in Figures 1b and 2a and Supplementary Figure 13a. Information about time between symptom onset and sample collection was unavailable for two samples from the mild COVID-19 group.

b) Linear regression between length of hospitalization in days and age is shown for severe and critical COVID-19 cases. Error bands of linear regression depicts 95% confidence interval.

c) Boxplot shows length of hospitalization in days for severe and critical COVID-19 cases. Two-sided Wilcoxon test was performed to determine significant difference between severe and critical COVID-19 cases. Boxplots depict median and interquartile range (IQR) and length of whiskers is 1.5 times IQR.

d) Length of hospitalization in days (x-axis) is shown as triangle and circles highlight length in days between symptom onset and sample collection. Donors are depicted in rows (y-axis). Symbols from hospitalized individuals are connected by colored bar. Blue or red bars highlight if length of hospitalization is shorter or longer, respectively. COVID-19 study groups based on severity are shown separately.

e) Length of hospitalization (grey bar), admission to ICU (red symbol) and sample collection (blue symbol) based on days from symptom onset is shown for severe and critical COVID-19 cases. Death is indicated by cross. Source data are provided as a Source Data file.

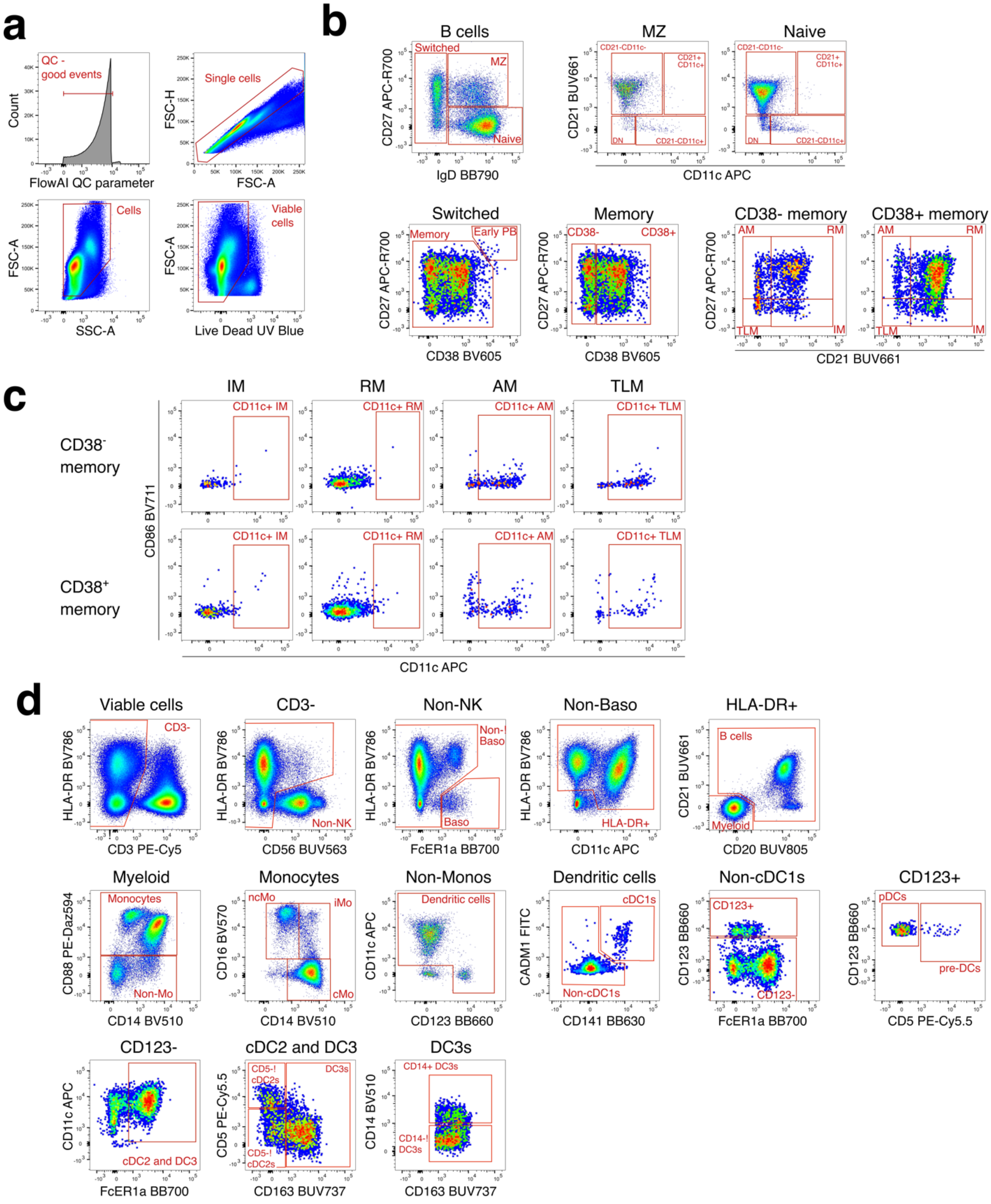
a
QC - good events
Count
FlowAI QC parameter
Single cells
FSC-H
FSC-A
Cells
FSC-A
SSC-A
Viable cells
Live Dead UV Blue
b
B cells
Switched
MZ
Naive
CD27 APC-R700
IgD BB790
MZ
Naive
CD21-CD11c-
CD21+ CD11c+
DN
CD21-CD11c+
CD21 BUV661
CD11c APC
Switched
Memory
Early PB
CD38 BV605
Memory
CD38-
CD38+
CD38- memory
CD38+ memory
AM
RM
TLM
IM
CD21 BUV661
c
IM
RM
AM
TLM
CD38- memory
CD38+ memory
CD11c+ IM
CD11c+ RM
CD11c+ AM
CD11c+ TLM
CD86 BV711
CD11c APC
d
Viable cells
CD3-
HLA-DR BV786
CD3 PE-Cy5
Non-NK
CD56 BUV563
Non-NK
Non-Baso
Baso
FcER1a BB700
Non-Baso
HLA-DR+
CD11c APC
HLA-DR+
B cells
Myeloid
CD21 BUV661
CD20 BUV805
Myeloid
Monocytes
Non-Mo
CD88 PE-Daz594
CD14 BV510
Monocytes
ncMo
iMo
cMo
CD16 BV570
Non-Monos
Dendritic cells
CD11c APC
CD123 BB660
Dendritic cells
cDC1s
Non-cDC1s
CADM1 FITC
CD141 BB630
Non-cDC1s
CD123+
CD123-
CD123 BB660
FcER1a BB700
CD123+
pDCs
pre-DCs
CD5 PE-Cy5.5
CD123-
cDC2 and DC3
CD11c APC
FcER1a BB700
cDC2 and DC3
DC3s
CD5 PE-Cy5.5
CD163 BUV737
DC3s
CD14+ DC3s
CD14 BV510
CD163 BUV737

Supplementary Figure 2: Gating of B cell subsets and myeloid cells (Monocytes/ Dendritic cells)

a) Strategy for subsequent removal of irregular events based on FlowAI (Upper left plot; FlowAI QC parameter), cell aggregates based on forward scatter height (FSC-H) and area (FSC-A) signal (Upper right plot), cell debris based on forward (FSC-A) and side (SSC-A) scatter (Lower left plot) and dead cells based on viability dye (Lower right plot; Live Dead UV Blue). **b)** Gating of B cell subsets and **c)** $CD11c^+$ B cells within memory B cell subsets (Intermediate memory, IM; Resting memory, RM; Activated memory, AM; Tissue-like memory, TLM) of $CD38^-$ (top row) and $CD38^+$ (bottom row) memory B cells is depicted. **d)** Gating of monocyte and dendritic cell subsets is shown.

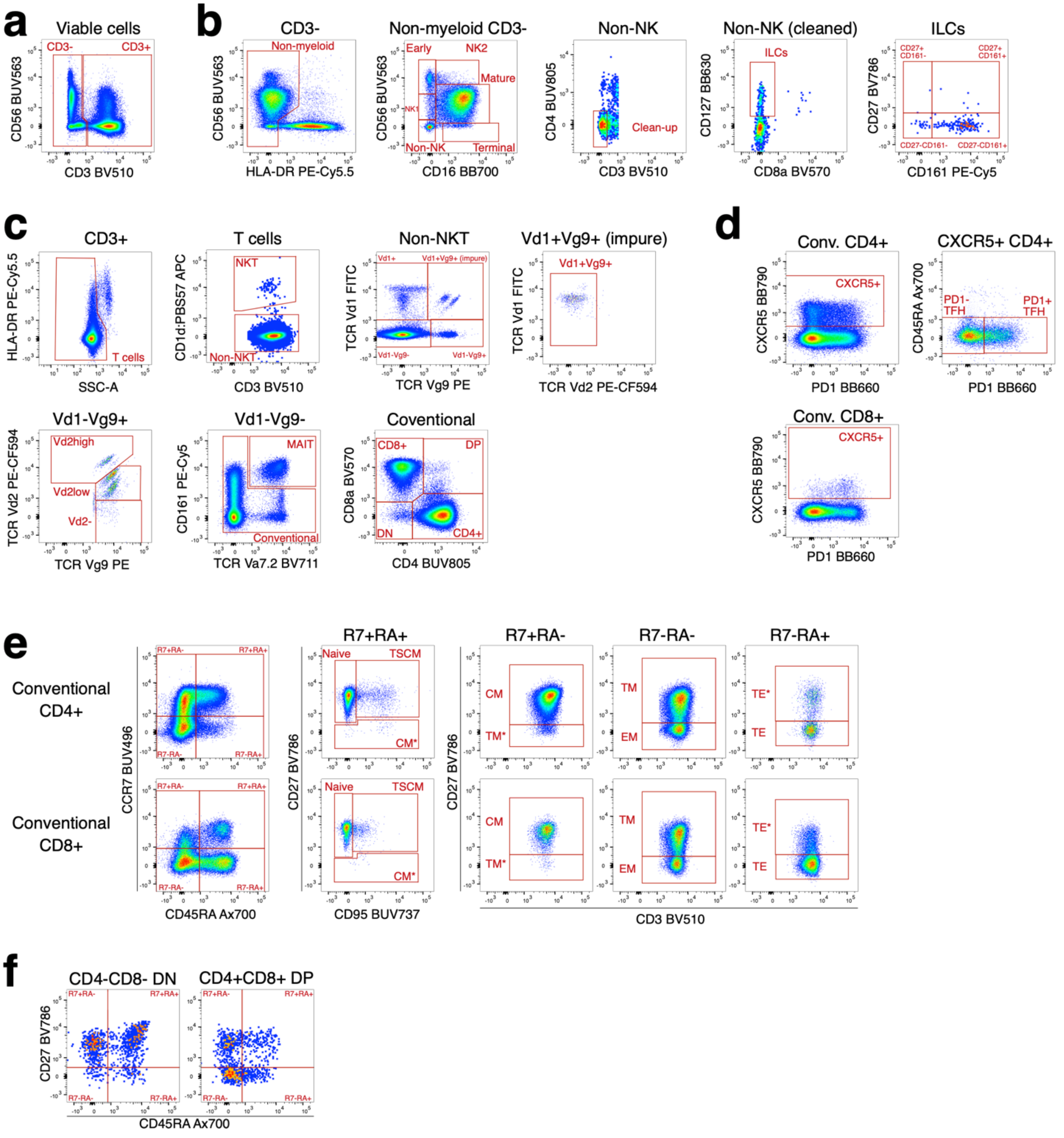
a
Viable cells
CD3-
CD3+
CD56 BUV563
CD3 BV510
b
CD3-
Non-myeloid
HLA-DR PE-Cy5.5
Non-myeloid CD3-
Early
NK2
Mature
NK1
Non-NK
Terminal
CD16 BB700
Non-NK
CD4 BUV805
Clean-up
Non-NK (cleaned)
ILCs
CD127 BB630
CD8a BV570
ILCs
CD27+ CD161-
CD27+ CD161+
CD27-CD161-
CD27-CD161+
CD27 BV786
CD161 PE-Cy5
c
CD3+
HLA-DR PE-Cy5.5
T cells
SSC-A
T cells
NKT
Non-NKT
CD1d:PBS57 APC
Non-NKT
Vd1+
Vd1+Vg9+ (impure)
Vd1-Vg9-
Vd1-Vg9+
TCR Vd1 FITC
TCR Vg9 PE
Vd1+Vg9+ (impure)
Vd1+Vg9+
TCR Vd2 PE-CF594
Vd1-Vg9+
Vd2high
Vd2low
Vd2-
Vd1-Vg9-
MAIT
Conventional
CD161 PE-Cy5
TCR Va7.2 BV711
Coventional
CD8+
DP
DN
CD4+
CD8a BV570
CD4 BUV805
d
Conv. CD4+
CXCR5+
CXCR5 BB790
PD1 BB660
CXCR5+ CD4+
PD1- TFH
PD1+ TFH
CD45RA Ax700
Conv. CD8+
CXCR5+
e
Conventional CD4+
Conventional CD8+
R7+RA-
R7+RA+
R7-RA-
R7-RA+
CCR7 BUV496
CD45RA Ax700
R7+RA+
Naive
TSCM
CM*
CD27 BV786
CD95 BUV737
R7+RA-
CM
TM*
R7-RA-
TM
EM
R7-RA+
TE*
TE
CD3 BV510
f
CD4-CD8- DN
CD4+CD8+ DP
CD27 BV786
CD45RA Ax700

Supplementary Figure 3: Gating of innate-like and conventional T cell subsets and NK cells

a) Definition of $CD3^-$ and $CD3^+$ cells is shown. **b)** Gating of NK cells and innate lymphoid cells (ILCs) is depicted. HLA-DR expressing cells were excluded prior to defining NK cells based on CD56 and CD16. We further removed residual contaminating cells within $CD56^-$ $CD16^-$ cells based on CD4 and CD8 prior to defining CD127 expressing ILCs and subsets of ILCs based on CD27 and CD161 expression. **c)** Definition of unconventional and conventional T cells is shown after excluding residual myeloid cells based on SSC-A and expression of HLA-DR. **d)** Gating of $CXCR5^+$ $CD4^+$ T cells and subsequent definition of follicular helper T cells (TFH) expressing or lacking PD-1 is shown as well as gating of CXCR5-expressing $CD8^+$ T cells. **e)** Definition of $CD4^+$ and $CD8^+$ T cell memory subsets is depicted. **f)** We further defined subsets from $CD4^-CD8^-$ double-negative (DN) and $CD4^+CD8^+$ double-positive (DP) T cells based on expression of CD27 and CD45RA.

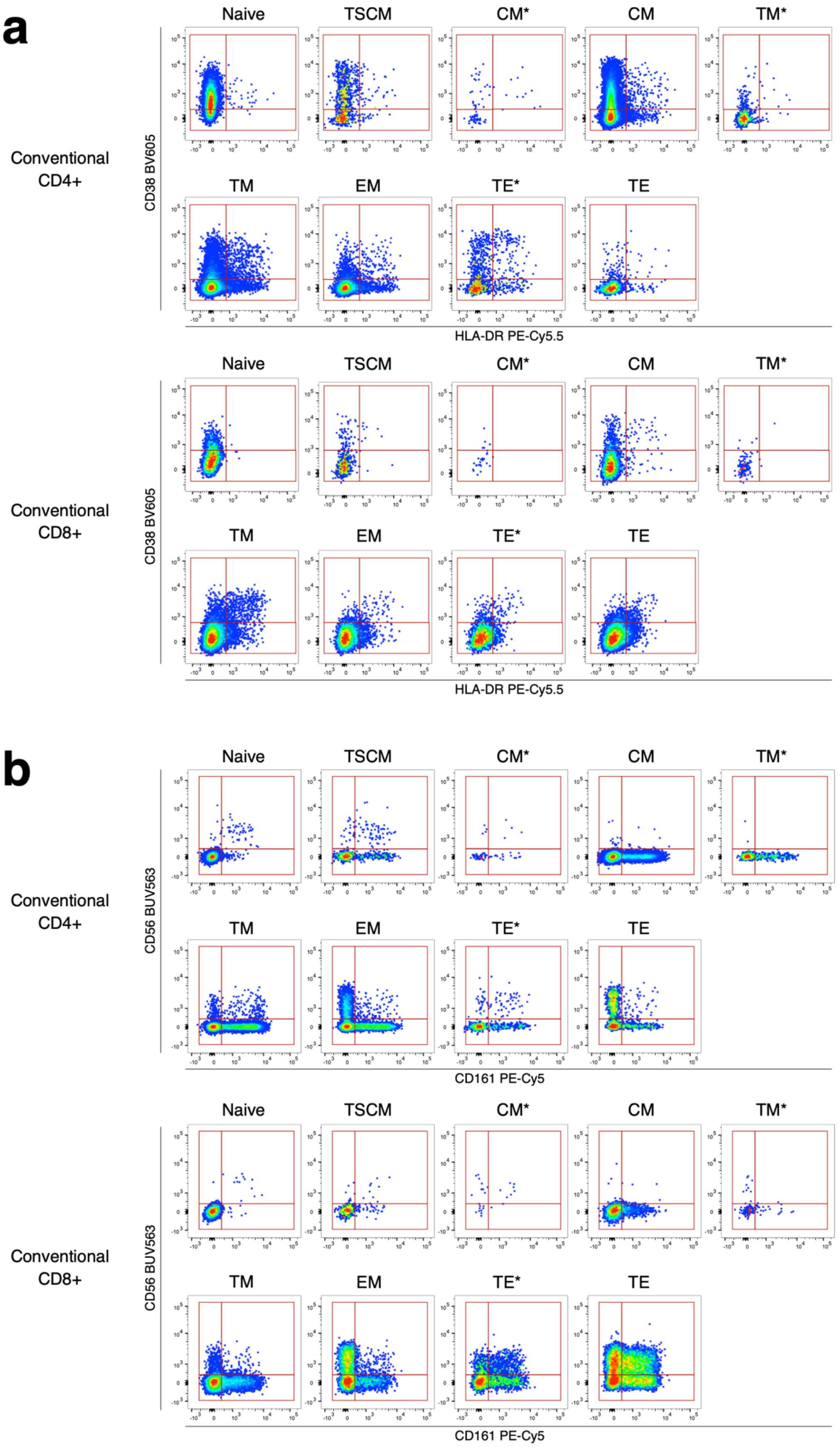
a
Conventional CD4+
Naive
TSCM
CM*
CM
TM*
TM
EM
TE*
TE
CD38 BV605
HLA-DR PE-Cy5.5
Conventional CD8+
b
CD56 BUV563
CD161 PE-Cy5

Supplementary Figure 4: Gating conventional T cell subsets based on CD38, HLA-DR, CD56 and CD161

Definition of subsets within conventional $CD4^+$ and $CD8^+$ naïve and memory T cell populations based on **a)** CD38 and HLA-DR or **b)** CD56 and CD161 expression.

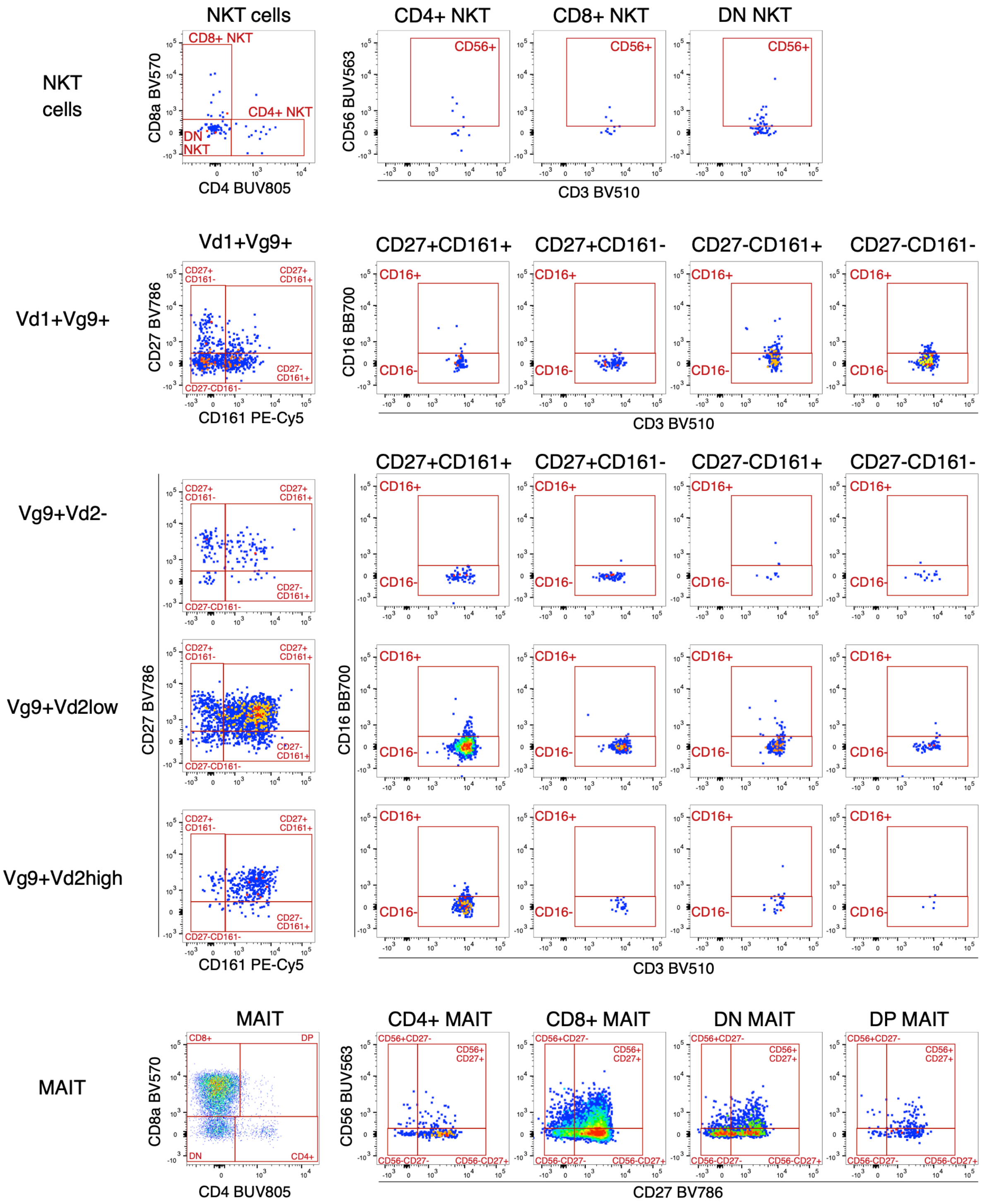

Supplementary Figure 5: Gating of several differentiation stages within innate-like T cell subsets

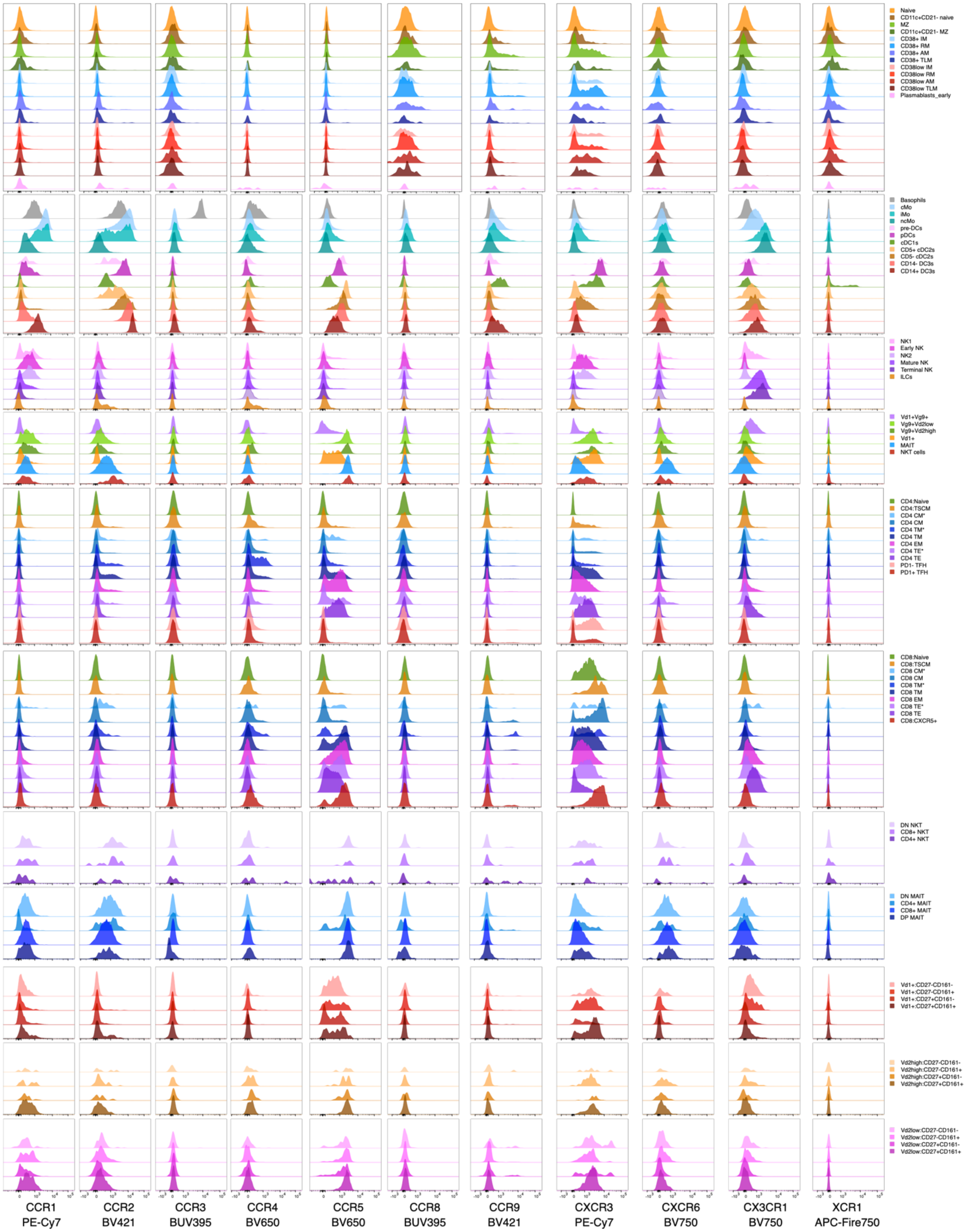

CCR1
PE-Cy7
CCR2
BV421
CCR3
BUV395
CCR4
BV650
CCR5
BV650
CCR8
BUV395
CCR9
BV421
CXCR3
PE-Cy7
CXCR6
BV750
CX3CR1
BV750
XCR1
APC-Fire750
Naive
CD11c+CD21- naive
MZ
CD11c+CD21- MZ
CD38+ IM
CD38+ RM
CD38+ AM
CD38+ TLM
CD38low IM
CD38low RM
CD38low AM
CD38low TLM
Plasmablasts_early
Basophils
cMo
iMo
ncMo
pre-DCs
pDCs
cDC1s
CD5+ cDC2s
CD5- cDC2s
CD14- DC3s
CD14+ DC3s
NK1
Early NK
NK2
Mature NK
Terminal NK
ILCs
Vd1+Vg9+
Vg9+Vd2low
Vg9+Vd2high
Vd1+
MAIT
NKT cells
CD4:Naive
CD4:TSCM
CD4 CM*
CD4 CM
CD4 TM*
CD4 TM
CD4 EM
CD4 TE*
CD4 TE
PD1- TFH
PD1+ TFH
CD8:Naive
CD8:TSCM
CD8 CM*
CD8 CM
CD8 TM*
CD8 TM
CD8 EM
CD8 TE*
CD8 TE
CD8:CXCR5+
DN NKT
CD8+ NKT
CD4+ NKT
DN MAIT
CD4+ MAIT
CD8+ MAIT
DP MAIT
Vd1+:CD27-CD161-
Vd1+:CD27-CD161+
Vd1+:CD27+CD161-
Vd1+:CD27+CD161+
Vd2high:CD27-CD161-
Vd2high:CD27-CD161+
Vd2high:CD27+CD161-
Vd2high:CD27+CD161+
Vd2low:CD27-CD161-
Vd2low:CD27-CD161+
Vd2low:CD27+CD161-
Vd2low:CD27+CD161+

Supplementary Figure 6: Expression of chemokine receptors

Shown is the expression of chemokine receptors on all main lineages/immune subsets as overlaid histograms. Data derives from one unexposed healthy donor.

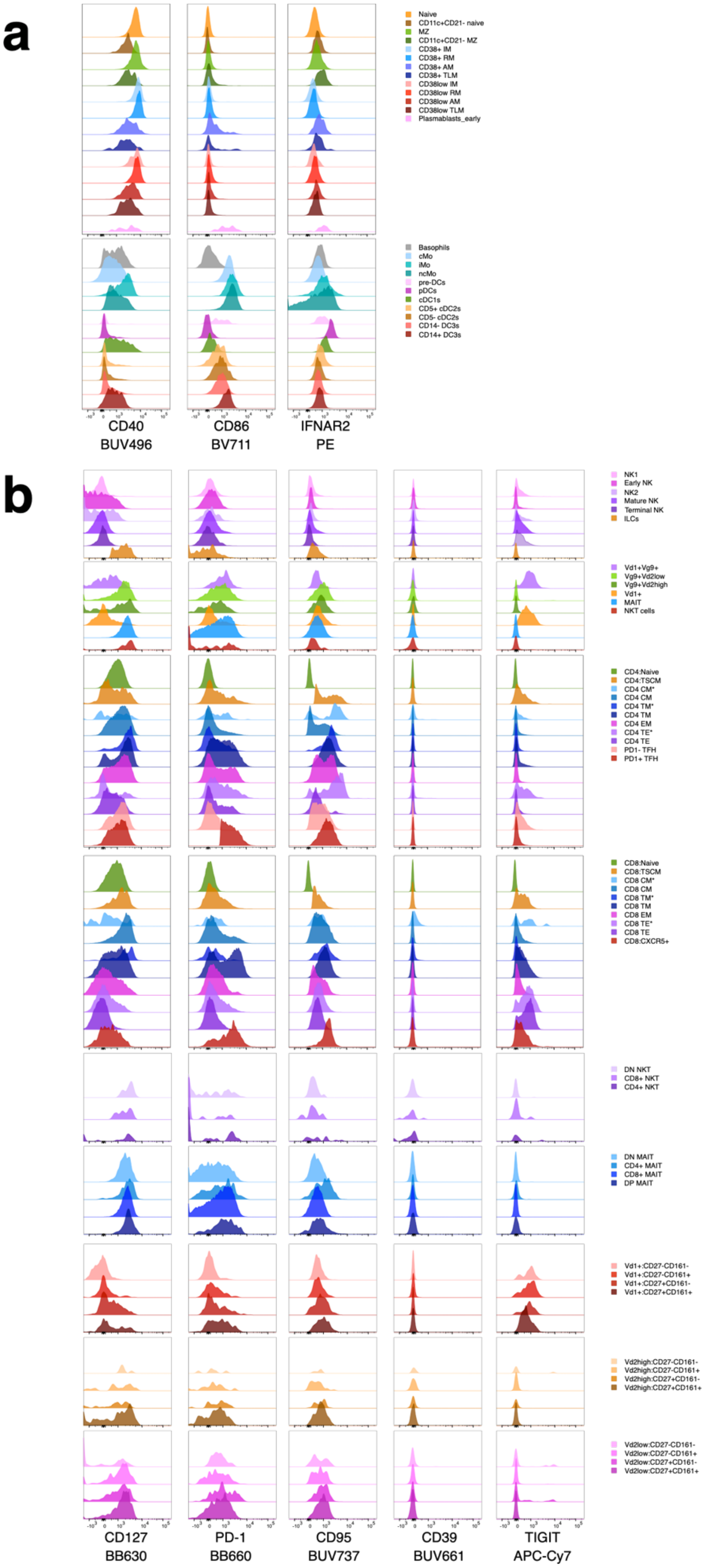
a
Naive
CD11c+CD21- naive
MZ
CD11c+CD21- MZ
CD38+ IM
CD38+ RM
CD38+ AM
CD38+ TLM
CD38low IM
CD38low RM
CD38low AM
CD38low TLM
Plasmablasts_early
Basophils
cMo
iMo
ncMo
pre-DCs
pDCs
cDC1s
CD5+ cDC2s
CD5- cDC2s
CD14- DC3s
CD14+ DC3s
CD40
BUV496
CD86
BV711
IFNAR2
PE
b
NK1
Early NK
NK2
Mature NK
Terminal NK
ILCs
Vd1+Vg9+
Vg9+Vd2low
Vg9+Vd2high
Vd1+
MAIT
NKT cells
CD4:Naive
CD4:TSCM
CD4 CM*
CD4 CM
CD4 TM*
CD4 TM
CD4 EM
CD4 TE*
CD4 TE
PD1- TFH
PD1+ TFH
CD8:Naive
CD8:TSCM
CD8 CM*
CD8 CM
CD8 TM*
CD8 TM
CD8 EM
CD8 TE*
CD8 TE
CD8:CXCR5+
DN NKT
CD8+ NKT
CD4+ NKT
DN MAIT
CD4+ MAIT
CD8+ MAIT
DP MAIT
Vd1+:CD27-CD161-
Vd1+:CD27-CD161+
Vd1+:CD27+CD161-
Vd1+:CD27+CD161+
Vd2high:CD27-CD161-
Vd2high:CD27-CD161+
Vd2high:CD27+CD161-
Vd2high:CD27+CD161+
Vd2low:CD27-CD161-
Vd2low:CD27-CD161+
Vd2low:CD27+CD161-
Vd2low:CD27+CD161+
CD127
BB630
PD-1
BB660
CD95
BUV737
CD39
BUV661
TIGIT
APC-Cy7

Supplementary Figure 7: Expression of functional receptors

Shown is the expression of functional receptors on main lineages/immune subsets as overlaid histograms. Markers are panel specific. Markers only measured with the **a)** B cell/myeloid cell or **b)** T cell/NK cell panel backbone are shown as highlighted in Supplementary Table 2. Data derives from one unexposed healthy donor.

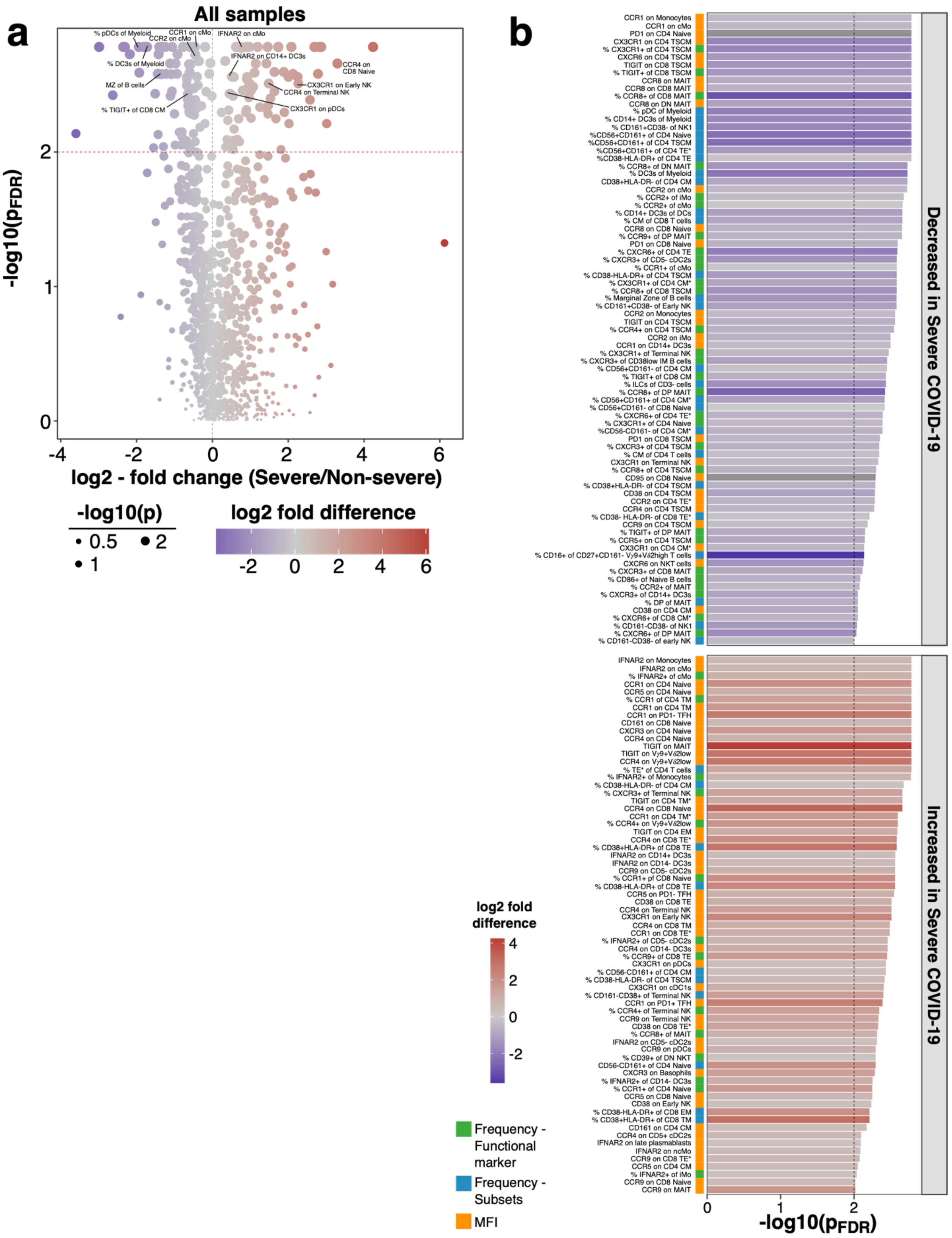
a
All samples
% pDCs of Myeloid
CCR1 on cMo
CCR2 on cMo
IFNAR2 on cMo
IFNAR2 on CD14+ DC3s
% DC3s of Myeloid
CCR4 on CD8 Naive
MZ of B cells
CX3CR1 on Early NK
CCR4 on Terminal NK
% TIGIT+ of CD8 CM
CX3CR1 on pDCs
-log10(pFDR)
log2 - fold change (Severe/Non-severe)
-log10(p)
0.5
1
2
log2 fold difference
b
CCR1 on Monocytes
CCR1 on cMo
PD1 on CD4 Naive
CX3CR1 on CD4 TSCM
% CX3CR1+ of CD4 TSCM
CXCR6 on CD4 TSCM
TIGIT on CD8 TSCM
% TIGIT+ of CD8 TSCM
CCR8 on MAIT
CCR8 on CD8 MAIT
% CCR8+ of CD8 MAIT
CCR8 on DN MAIT
% pDC of Myeloid
% CD14+ DC3s of Myeloid
% CD161+CD38- of NK1
%CD56+CD161+ of CD4 Naive
%CD56+CD161+ of CD4 TSCM
%CD56+CD161+ of CD4 TE*
%CD38-HLA-DR+ of CD4 TE
% CCR8+ of DN MAIT
% DC3s of Myeloid
CD38+HLA-DR- of CD4 CM
CCR2 on cMo
% CCR2+ of iMo
% CCR2+ of cMo
% CD14+ DC3s of DCs
% CM of CD8 T cells
CCR8 on CD8 Naive
% CCR9+ of DP MAIT
PD1 on CD8 Naive
% CXCR6+ of CD4 TE
% CXCR3+ of CD5- cDC2s
% CCR1+ of cMo
% CD38-HLA-DR+ of CD4 TSCM
% CX3CR1+ of CD4 CM*
% CCR8+ of CD8 TSCM
% Marginal Zone of B cells
% CD161+CD38- of Early NK
CCR2 on Monocytes
TIGIT on CD4 TSCM
% CCR4+ on CD4 TSCM
CCR2 on iMo
CCR1 on CD14+ DC3s
% CX3CR1+ of Terminal NK
% CXCR3+ of CD38low IM B cells
% CD56+CD161- of CD4 CM
% TIGIT+ of CD8 CM
% ILCs of CD3- cells
% CCR8+ of DP MAIT
% CD56+CD161+ of CD4 CM*
% CD56+CD161- of CD8 Naive
% CXCR6+ of CD4 TE*
% CX3CR1+ of CD4 Naive
%CD56-CD161- of CD4 CM*
PD1 on CD8 TSCM
% CXCR3+ of CD4 TSCM
% CM of CD4 T cells
CX3CR1 on Terminal NK
% CCR8+ of CD4 TSCM
CD95 on CD8 Naive
% CD38+HLA-DR- of CD4 TSCM
CD38 on CD4 TSCM
CCR2 on CD4 TE*
CCR4 on CD4 TSCM
% CD38- HLA-DR- of CD8 TE*
CCR9 on CD4 TSCM
% TIGIT+ of DP MAIT
% CCR5+ on CD4 TSCM
CX3CR1 on CD4 CM*
% CD16+ of CD27+CD161- Vγ9+Vδ2high T cells
CXCR6 on NKT cells
% CXCR3+ of CD8 MAIT
% CD86+ of Naive B cells
% CCR2+ of MAIT
% CXCR3+ of CD14+ DC3s
% DP of MAIT
CD38 on CD4 CM
% CXCR6+ of CD8 CM*
% CD161-CD38- of NK1
% CXCR6+ of DP MAIT
% CD161-CD38- of early NK
Decreased in Severe COVID-19
IFNAR2 on Monocytes
IFNAR2 on cMo
% IFNAR2+ of cMo
CCR1 on CD4 Naive
CCR5 on CD4 Naive
% CCR1 of CD4 TM
CCR1 on CD4 TM
CCR1 on PD1- TFH
CD161 on CD8 Naive
CXCR3 on CD4 Naive
CCR4 on CD4 Naive
TIGIT on MAIT
TIGIT on Vγ9+Vδ2low
CCR4 on Vγ9+Vδ2low
% TE* of CD4 T cells
% IFNAR2+ of Monocytes
% CD38-HLA-DR- of CD4 CM
% CXCR3+ of Terminal NK
TIGIT on CD4 TM*
CCR4 on CD8 Naive
CCR1 on CD4 TM*
% CCR4+ on Vγ9+Vδ2low
TIGIT on CD4 EM
CCR4 on CD8 TE*
% CD38+HLA-DR+ of CD8 TE
IFNAR2 on CD14+ DC3s
IFNAR2 on CD14- DC3s
CCR9 on CD5- cDC2s
% CCR1+ pf CD8 Naive
% CD38-HLA-DR+ of CD8 TE
CCR5 on PD1- TFH
CD38 on CD8 TE
CCR4 on Terminal NK
CX3CR1 on Early NK
CCR4 on CD8 TM
CCR1 on CD8 TE*
% IFNAR2+ of CD5- cDC2s
CCR4 on CD14- DC3s
% CCR9+ of CD8 TE
CX3CR1 on pDCs
% CD56-CD161+ of CD4 CM
% CD38-HLA-DR- of CD4 TSCM
CX3CR1 on cDC1s
% CD161-CD38+ of Terminal NK
CCR1 on PD1+ TFH
% CCR4+ of Terminal NK
CCR9 on Terminal NK
CD38 on CD8 TE*
% CCR8+ of MAIT
IFNAR2 on CD5- cDC2s
CCR9 on pDCs
% CD39+ of DN NKT
CD56-CD161+ of CD4 Naive
CXCR3 on Basophils
% IFNAR2+ of CD14- DC3s
% CCR1+ of CD4 Naive
CCR5 on CD8 Naive
CD38 on Early NK
% CD38-HLA-DR+ of CD8 EM
% CD38+HLA-DR+ of CD8 TM
CD161 on CD4 CM
CCR4 on CD5+ cDC2s
IFNAR2 on late plasmablasts
IFNAR2 on ncMo
CCR9 on CD8 TE*
CCR5 on CD4 CM
% IFNAR2+ of iMo
CCR9 on CD8 Naive
CCR9 on MAIT
Increased in Severe COVID-19
-log10(pFDR)
log2 fold difference
Frequency - Functional marker
Frequency - Subsets
MFI

Supplementary Figure 8: Comparison of individuals recovered from non-severe and severe COVID-19

a) Volcano plot shows comparison of individuals recovered from non-severe (mild/moderate) and severe (severe/critical) COVID-19. P-values were obtained from logistic regression, included correction for age and experiment and were corrected for multiple testing using Benjamini-Hochberg false discovery rate. Log2 fold change was calculated based on the mean of immune traits within non-severe and severe COVID-19 cases. P-values are shown as -log10.

b) Bar graph shows FDR-adjusted -log10 P-values for significant immune traits with $P < 0.01$ derived from Supplementary Figure 8a. Bars are colored based on log2 fold change and split based on decrease (top) or increase (bottom) in individuals recovered from severe COVID-19. Bar on the left indicates trait type. Source data are provided as a Source Data file.

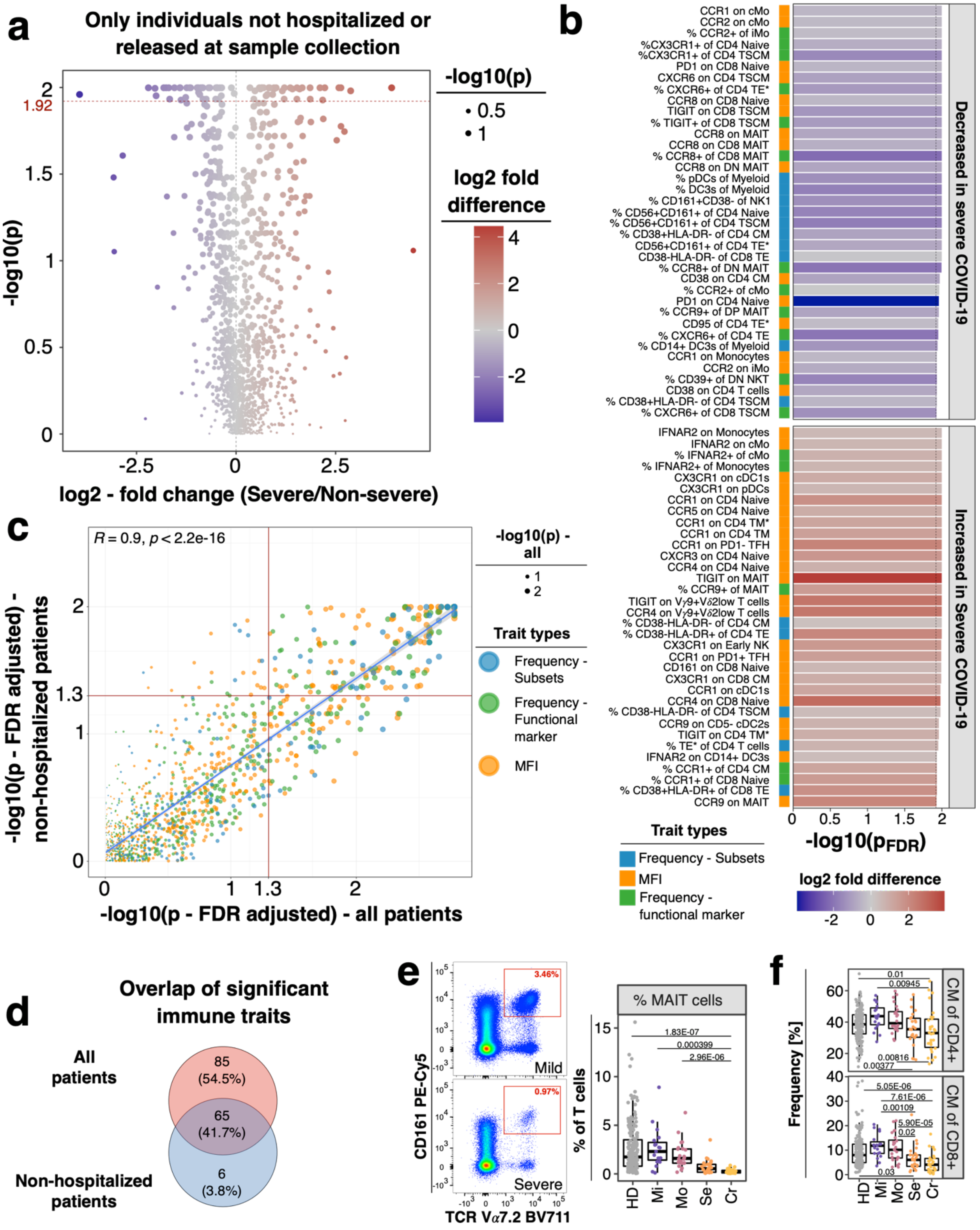

a
Only individuals not hospitalized or released at sample collection
-log10(p)
log2 - fold change (Severe/Non-severe)
1.92
-log10(p)
0.5
1
log2 fold difference
b
CCR1 on cMo
CCR2 on cMo
% CCR2+ of iMo
%CX3CR1+ of CD4 Naive
%CX3CR1+ of CD4 TSCM
PD1 on CD8 Naive
CXCR6 on CD4 TSCM
% CXCR6+ of CD4 TE*
CCR8 on CD8 Naive
TIGIT on CD8 TSCM
% TIGIT+ of CD8 TSCM
CCR8 on MAIT
CCR8 on CD8 MAIT
% CCR8+ of CD8 MAIT
CCR8 on DN MAIT
% pDCs of Myeloid
% DC3s of Myeloid
% CD161+CD38- of NK1
% CD56+CD161+ of CD4 Naive
% CD56+CD161+ of CD4 TSCM
% CD38+HLA-DR- of CD4 CM
CD56+CD161+ of CD4 TE*
CD38-HLA-DR- of CD8 TE
% CCR8+ of DN MAIT
CD38 on CD4 CM
% CCR2+ of cMo
PD1 on CD4 Naive
% CCR9+ of DP MAIT
CD95 of CD4 TE*
% CXCR6+ of CD4 TE
% CD14+ DC3s of Myeloid
CCR1 on Monocytes
CCR2 on iMo
% CD39+ of DN NKT
CD38 on CD4 T cells
% CD38+HLA-DR- of CD4 TSCM
% CXCR6+ of CD8 TSCM
Decreased in severe COVID-19
IFNAR2 on Monocytes
IFNAR2 on cMo
% IFNAR2+ of cMo
% IFNAR2+ of Monocytes
CX3CR1 on cDC1s
CX3CR1 on pDCs
CCR1 on CD4 Naive
CCR5 on CD4 Naive
CCR1 on CD4 TM*
CCR1 on CD4 TM
CCR1 on PD1- TFH
CXCR3 on CD4 Naive
CCR4 on CD4 Naive
TIGIT on MAIT
% CCR9+ of MAIT
TIGIT on Vγ9+Vδ2low T cells
CCR4 on Vγ9+Vδ2low T cells
% CD38-HLA-DR- of CD4 CM
% CD38-HLA-DR+ of CD4 TE
CX3CR1 on Early NK
CCR1 on PD1+ TFH
CD161 on CD8 Naive
CX3CR1 on CD8 CM
CCR1 on cDC1s
CCR4 on CD8 Naive
% CD38-HLA-DR- of CD4 TSCM
CCR9 on CD5- cDC2s
TIGIT on CD4 TM*
% TE* of CD4 T cells
IFNAR2 on CD14+ DC3s
% CCR1+ of CD4 CM
% CCR1+ of CD8 Naive
% CD38+HLA-DR+ of CD8 TE
CCR9 on MAIT
Increased in Severe COVID-19
-log10(pFDR)
Trait types
Frequency - Subsets
MFI
Frequency - functional marker
log2 fold difference
c
R = 0.9, p < 2.2e-16
-log10(p - FDR adjusted) - non-hospitalized patients
-log10(p - FDR adjusted) - all patients
-log10(p) - all
Trait types
Frequency - Subsets
Frequency - Functional marker
MFI
d
Overlap of significant immune traits
All patients
85 (54.5%)
65 (41.7%)
6 (3.8%)
Non-hospitalized patients
e
3.46%
Mild
0.97%
Severe
CD161 PE-Cy5
TCR Vα7.2 BV711
% MAIT cells
% of T cells
1.83E-07
0.000399
2.96E-06
HD
Mi
Mo
Se
Cr
f
Frequency [%]
CM of CD4+
CM of CD8+
0.01
0.00945
0.00816
0.00377
5.05E-06
7.61E-06
0.00109
5.90E-05
0.02
0.03

Supplementary Figure 9: Comparison of analysis between all and non-hospitalized individuals at time of sample collection

a) Volcano plot shows comparison of individuals recovered from non-severe (mild/moderate) and severe (severe/critical) COVID-19. Only individuals not hospitalized or discharged at day of sample collection are included. P-values were obtained from logistic regression, included correction for age and experiment and were corrected for multiple testing using Benjamini-Hochberg false discovery rate. Log2 fold change was calculated based on the mean of immune traits within non-severe and severe COVID-19 cases. P-values are shown as -log10.

b) Bar graph shows FDR-adjusted -log10 P-values from logistic regression analysis for immune traits significantly different between non-severe and severe COVID-19 cases (cut-off for P-value < 0.012). Plot is similar to Supplementary Figure 8b but depicts P-values obtained with only individuals not hospitalized or released at day of sample collection. Bar on the left indicates the immune trait type. Color of bars indicate log2 fold change between non-severe and severe COVID-19 cases calculated as the ratio between the mean of immune traits between the two severity groups.

c) Comparison of stable immune traits between non-severe and severe COVID-19 cases including either all individuals (x-axis) or only individuals not hospitalized or released at day of sample collection (y-axis) is shown. Plot shows FDR-adjusted -log10 P-values for manually gated immune traits (N = 1365). P-values were obtained by logistic regression and corrected for age and experiment batch. Size of symbols is based on -log10 P-values from analysis including all individuals. Color depicts the type of trait.

d) Venn graph depicts overlap of immune traits which differed between non-severe and severe COVID-19 group obtained from analysis including all (red circle, traits from Supplementary Fig. 8b) or only non-hospitalized individuals (blue circle, traits from Supplementary Fig. 9b).

e) Example flow cytometry data and gating of MAIT cells is shown (left) for one donor recovered from mild and severe COVID-19. Boxplot (right) shows frequency of MAIT cells per group. More detailed gating information is shown in Supplementary Figure 3.

f) Boxplots show frequencies of CD4 (top) and CD8 (bottom) central memory (CM) cells of conventional CD4 and CD8 T cells, respectively, from all study groups. Residuals from

linear regression between immune trait and age were used to calculate statistics on age-corrected data. ANOVA with subsequent two-sided Wilcoxon test and Bonferroni correction on residuals was performed for statistics highlighted in boxplots. Boxplots depict median and interquartile range (IQR) and length of whiskers is 1.5 times IQR. Study groups encompass healthy unexposed controls (HD) and individuals recovered from mild (Mi), moderate (Mo), severe (Se) and critical (Cr) COVID-19. Source data are provided as a Source Data file.

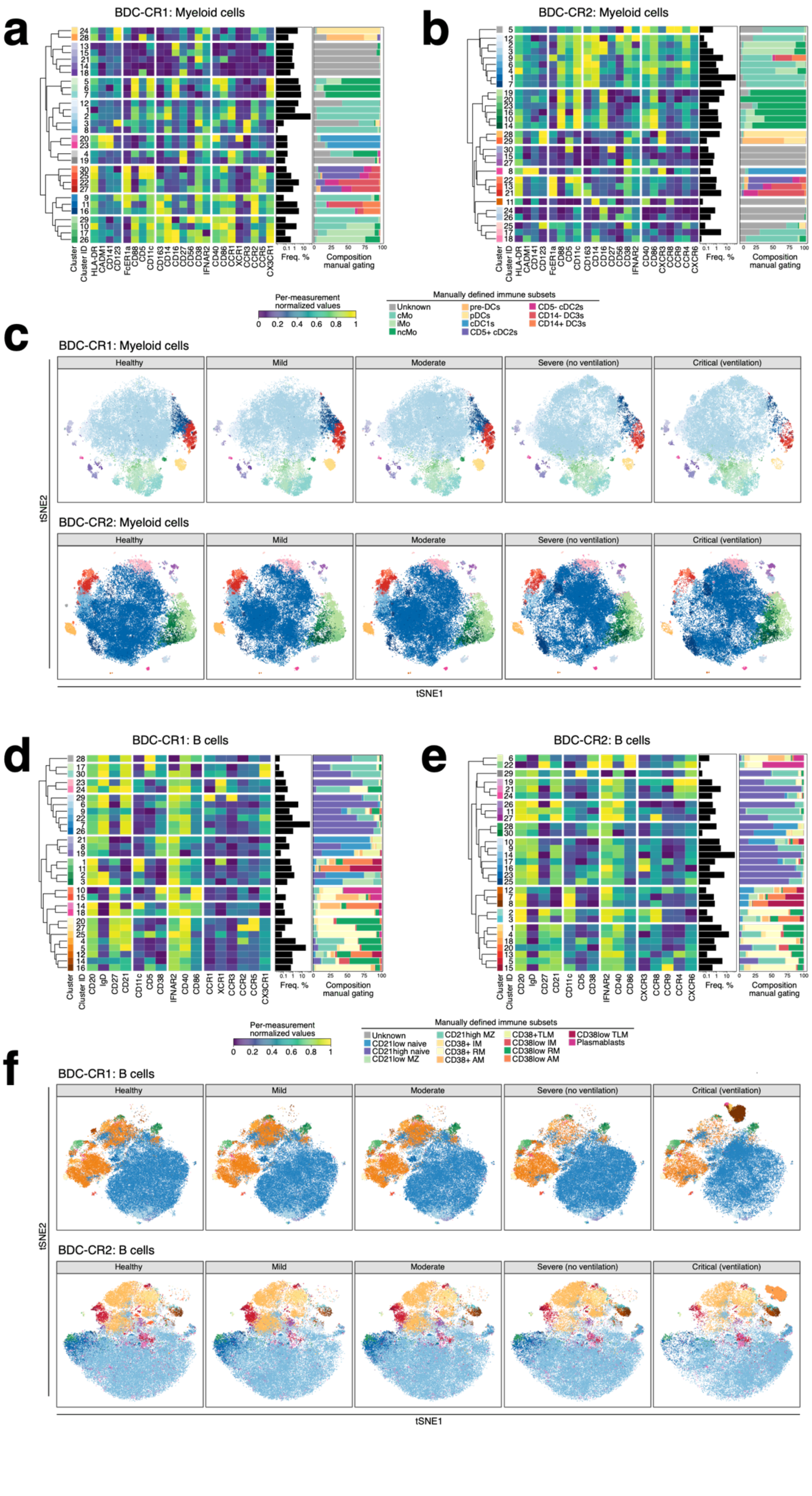
a
BDC-CR1: Myeloid cells
b
BDC-CR2: Myeloid cells
Cluster
Cluster ID
Freq. %
Composition manual gating
Per-measurement normalized values
0 0.2 0.4 0.6 0.8 1
Manually defined immune subsets
Unknown
cMo
iMo
ncMo
pre-DCs
pDCs
cDC1s
CD5+ cDC2s
CD5- cDC2s
CD14- DC3s
CD14+ DC3s
c
BDC-CR1: Myeloid cells
Healthy
Mild
Moderate
Severe (no ventilation)
Critical (ventilation)
BDC-CR2: Myeloid cells
tSNE2
tSNE1
d
BDC-CR1: B cells
e
BDC-CR2: B cells
Manually defined immune subsets
Unknown
CD21low naive
CD21high naive
CD21low MZ
CD21high MZ
CD38+ IM
CD38+ RM
CD38+ AM
CD38+TLM
CD38low IM
CD38low RM
CD38low AM
CD38low TLM
Plasmablasts
f
BDC-CR1: B cells
BDC-CR2: B cells

Supplementary Figure 10: FlowSOM analysis for myeloid cells and B cells

Heatmaps depict marker expression levels of myeloid cell (HLA-DR^{+}CD20^{-}) clusters derived from panels **a)** CR1 and **b)** CR2. **c)** tSNE plots for myeloid cells from CR1 (top) and CR2 (bottom) panel are shown delineated based on COVID-19 severity group. Similarly, heatmaps for **d)** CR1 and **e)** CR2 panels and **f)** tSNE analysis for B cells is shown. Heatmaps show per-measurement normalized median fluorescence intensity based on trimmed 1-99% percentile values for each FlowSOM cluster (rows). Only markers included in clustering (columns) are shown. Bar on left shows coloring of each FlowSOM cluster and FlowSOM clusters were clustered based on similarity of MFI values using hierarchical clustering (indicated by dendrogram and gap between rows). Bar graph in the middle shows the frequency of each cluster and bar graph on the right the composition of each cluster based on manual gating annotation. Dots in tSNE maps are colored based on FlowSOM cluster annotation. Each tSNE plot contains 50’000 randomly subsampled cells and not equally distributed across each individual sample.

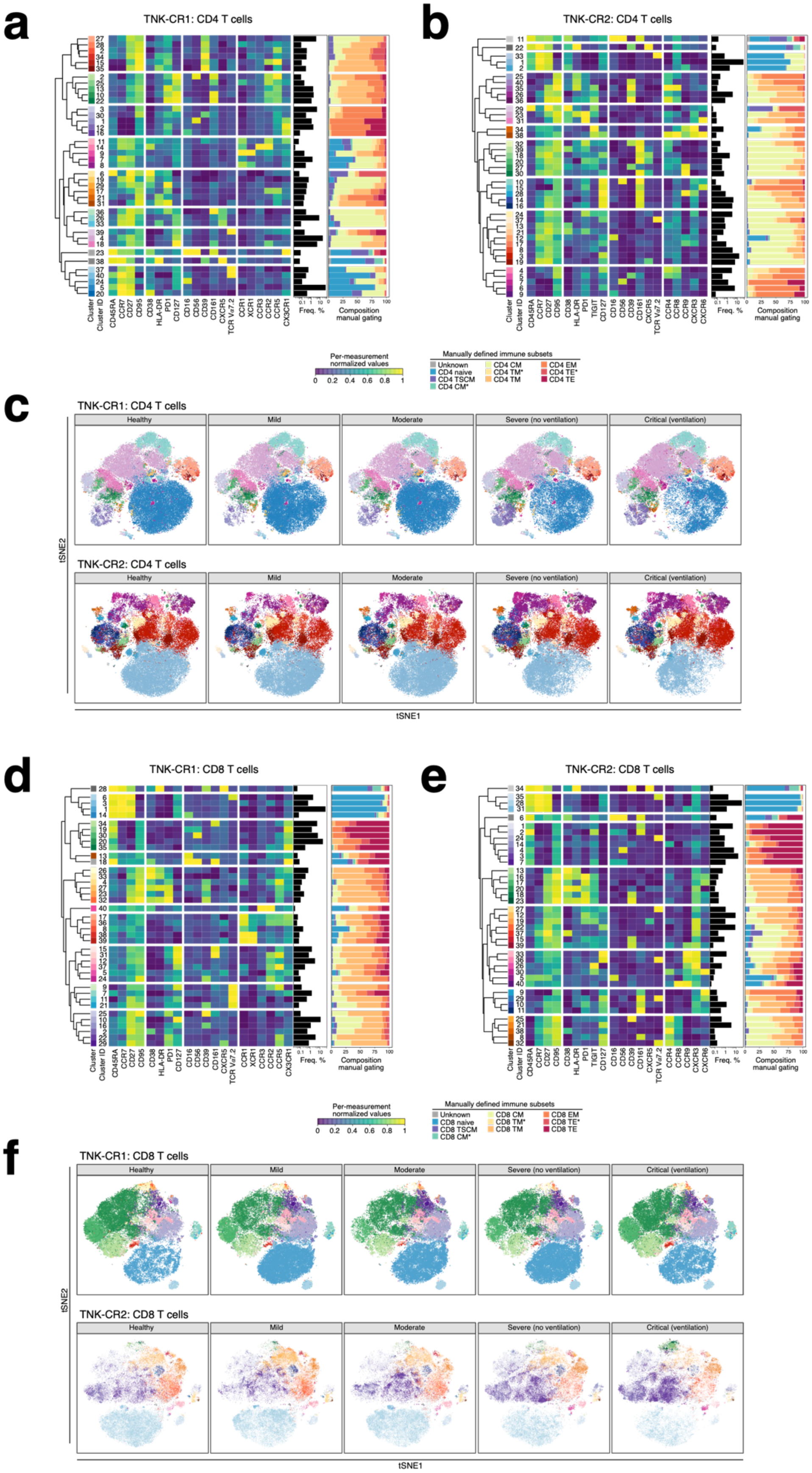
a
TNK-CR1: CD4 T cells
b
TNK-CR2: CD4 T cells
Cluster
Cluster ID
Freq. %
Composition manual gating
Per-measurement normalized values
0 0.2 0.4 0.6 0.8 1
Manually defined immune subsets
Unknown
CD4 naive
CD4 TSCM
CD4 CM*
CD4 CM
CD4 TM*
CD4 TM
CD4 EM
CD4 TE*
CD4 TE
c
TNK-CR1: CD4 T cells
Healthy
Mild
Moderate
Severe (no ventilation)
Critical (ventilation)
TNK-CR2: CD4 T cells
tSNE2
tSNE1
d
TNK-CR1: CD8 T cells
e
TNK-CR2: CD8 T cells
Unknown
CD8 naive
CD8 TSCM
CD8 CM*
CD8 CM
CD8 TM*
CD8 TM
CD8 EM
CD8 TE*
CD8 TE
f
TNK-CR1: CD8 T cells
TNK-CR2: CD8 T cells

Supplementary Figure 11: FlowSOM analysis for CD4 and CD8 T cells

Same as Supplementary Figure 10.

187

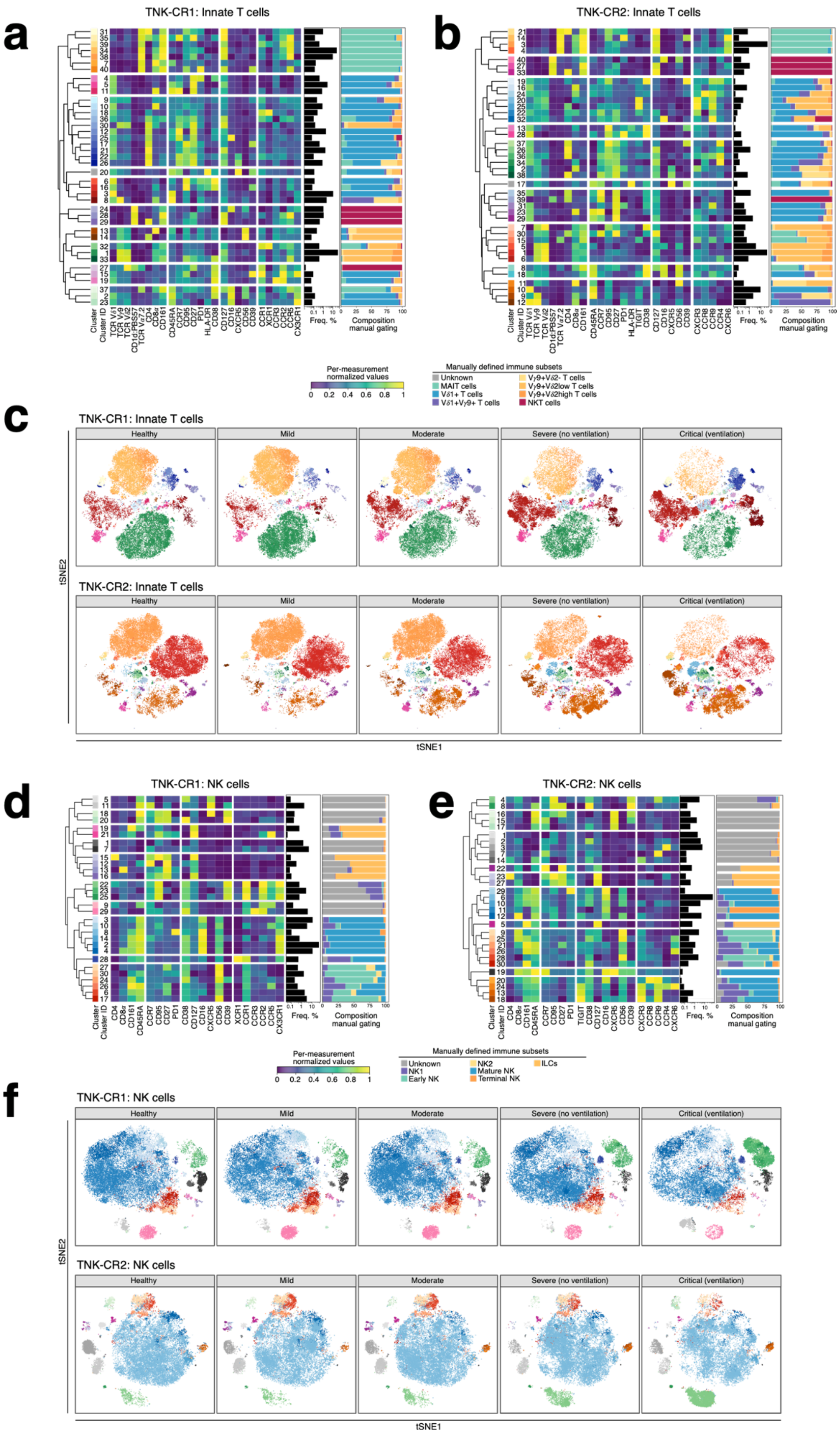

a
TNK-CR1: Innate T cells
b
TNK-CR2: Innate T cells
Freq. %
Composition manual gating
Per-measurement normalized values
Manually defined immune subsets
Unknown
MAIT cells
Vδ1+ T cells
Vδ1+Vγ9+ T cells
Vγ9+Vδ2- T cells
Vγ9+Vδ2low T cells
Vγ9+Vδ2high T cells
NKT cells
c
TNK-CR1: Innate T cells
TNK-CR2: Innate T cells
Healthy
Mild
Moderate
Severe (no ventilation)
Critical (ventilation)
tSNE1
tSNE2
d
TNK-CR1: NK cells
e
TNK-CR2: NK cells
Unknown
NK1
Early NK
NK2
Mature NK
Terminal NK
ILCs
f
TNK-CR1: NK cells
TNK-CR2: NK cells

Supplementary Figure 12: FlowSOM analysis for innate-like T cells and NK cells

Same as Supplementary Figure 10. For tSNE computation, 27583 and 25000 cells from each group were included for innate T cells and NK cells, respectively.

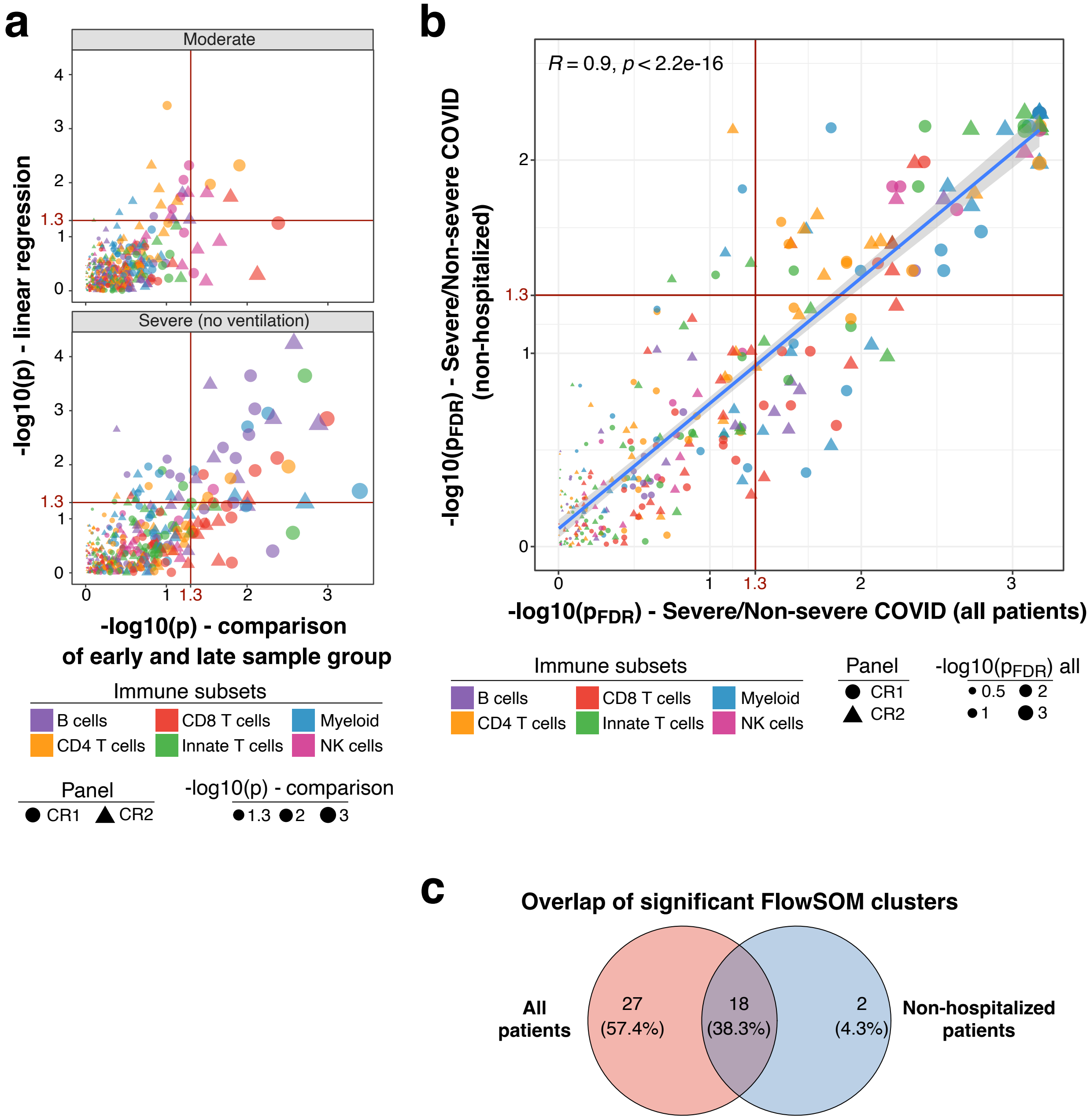
a
Moderate
Severe (no ventilation)
-log10(p) - linear regression
-log10(p) - comparison of early and late sample group
1.3
Immune subsets
B cells
CD4 T cells
CD8 T cells
Innate T cells
Myeloid
NK cells
Panel
CR1
CR2
-log10(p) - comparison
1.3
2
3
b
R = 0.9, p < 2.2e-16
-log10(pFDR) - Severe/Non-severe COVID (non-hospitalized)
-log10(pFDR) - Severe/Non-severe COVID (all patients)
1.3
Immune subsets
B cells
CD4 T cells
CD8 T cells
Innate T cells
Myeloid
NK cells
Panel
CR1
CR2
-log10(pFDR) all
0.5
1
2
3
c
Overlap of significant FlowSOM clusters
All patients
27 (57.4%)
18 (38.3%)
2 (4.3%)
Non-hospitalized patients

Supplementary Figure 13: Dynamics of FlowSOM clusters in COVID-19

a) FlowSOM clusters affected by long-term perturbations were identified in individuals recovered from moderate (top) or severe COVID-19 (bottom) either by linear regression of cluster frequency and days between symptom onset and sample collection or Wilcoxon analysis of cluster frequency between early and late timepoints (cut-off >60 days between symptom onset and sample collection). -log10 P-values from both analyses are shown for All 388 FlowSOM clusters. P-value cutoff of 0.05 is shown by red line. Symbols are colored based on lineage and shaped based on CR1 (circle) or CR2 (triangle) panel. Symbol size is according to -log10 P-value from Wilcoxon analysis.

b) Graph shows FDR-adjusted -log10 P-values derived from comparison of stable FlowSOM cluster (N = 291) frequencies between individuals recovered from non-severe and severe COVID-19. Analyses included either all individuals (x-axis) and only individuals not hospitalized or released at day of sample collection (y-axis). P-values were obtained by logistic regression correcting for age and experiment batch. Symbols are colored based on lineage and shape corresponds to CR1 or CR2 panel. Symbol size is based on FDR-adjusted -log10 P-value derived from analysis with all individuals.

c) Venn graph shows overlap of significant FlowSOM clusters between individuals recovered from non-severe and severe COVID-19 from analysis including either all individuals (red circle) or only individuals not hospitalized or released at day of sample collection (blue circle).

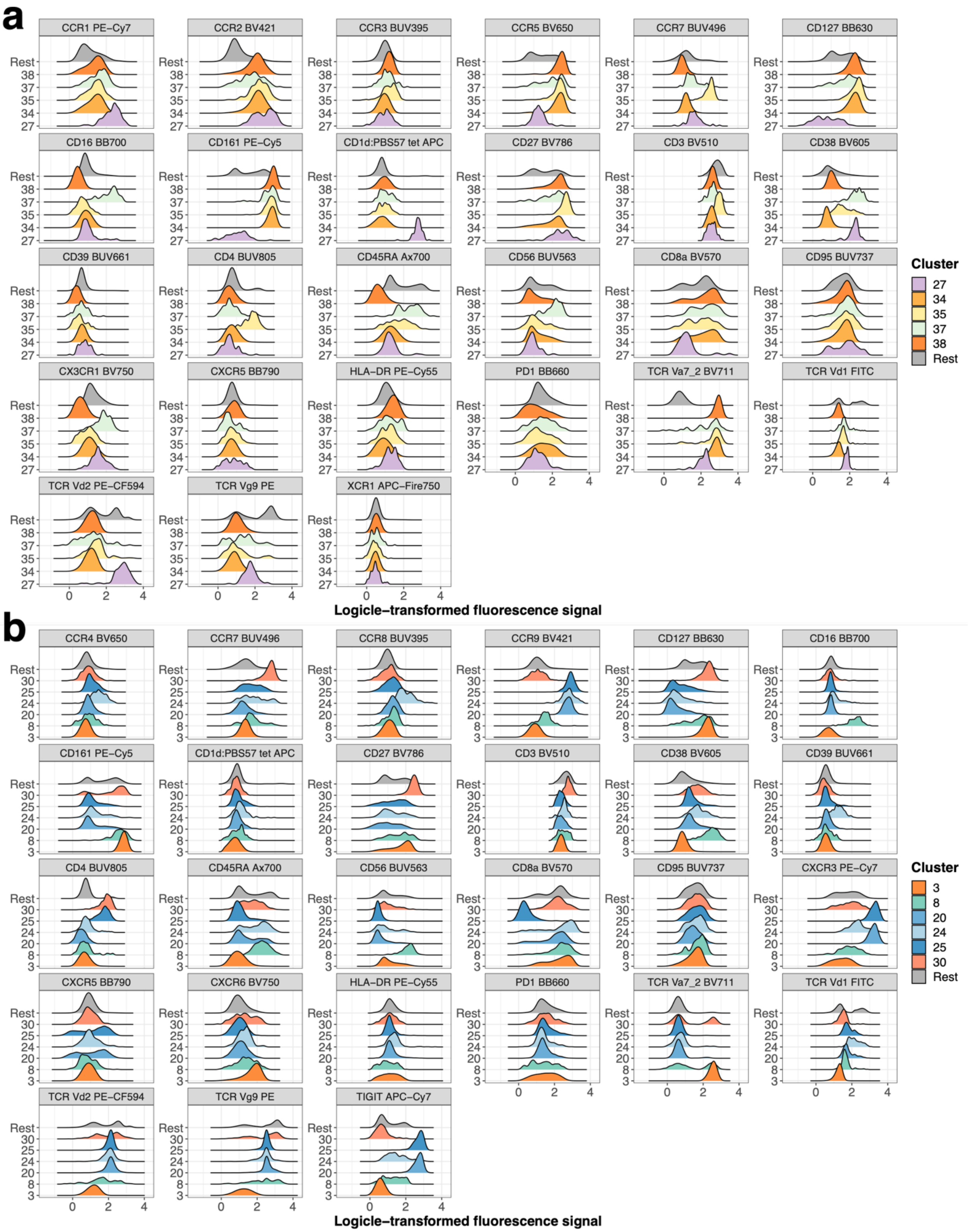
a
CCR1 PE−Cy7
CCR2 BV421
CCR3 BUV395
CCR5 BV650
CCR7 BUV496
CD127 BB630
CD16 BB700
CD161 PE−Cy5
CD1d:PBS57 tet APC
CD27 BV786
CD3 BV510
CD38 BV605
CD39 BUV661
CD4 BUV805
CD45RA Ax700
CD56 BUV563
CD8a BV570
CD95 BUV737
CX3CR1 BV750
CXCR5 BB790
HLA−DR PE−Cy55
PD1 BB660
TCR Va7_2 BV711
TCR Vd1 FITC
TCR Vd2 PE−CF594
TCR Vg9 PE
XCR1 APC−Fire750
Rest
38
37
35
34
27
Cluster
27
34
35
37
38
Rest
0
2
4
Logicle−transformed fluorescence signal
b
CCR4 BV650
CCR7 BUV496
CCR8 BUV395
CCR9 BV421
CD127 BB630
CD16 BB700
CD161 PE−Cy5
CD1d:PBS57 tet APC
CD27 BV786
CD3 BV510
CD38 BV605
CD39 BUV661
CD4 BUV805
CD45RA Ax700
CD56 BUV563
CD8a BV570
CD95 BUV737
CXCR3 PE−Cy7
CXCR5 BB790
CXCR6 BV750
HLA−DR PE−Cy55
PD1 BB660
TCR Va7_2 BV711
TCR Vd1 FITC
TCR Vd2 PE−CF594
TCR Vg9 PE
TIGIT APC−Cy7
Rest
30
25
24
20
8
3
Cluster
3
8
20
24
25
30
Rest
0
2
4
Logicle−transformed fluorescence signal

Supplementary Figure 14: Expression pattern of significant innate-like T cell clusters between non-severe and severe COVID-19

Expression (logicle-transformed fluorescence signal) of markers from **a)** CR1 and **b)** CR2 panel for innate-like T cell clusters are shown as overlaid histograms. T cell clusters are described in Figure 5c and d and are significantly different between individuals recovered from non-severe and severe COVID-19. All remaining clusters within innate-like T cells are depicted in grey and labeled as “Rest” as a reference population.

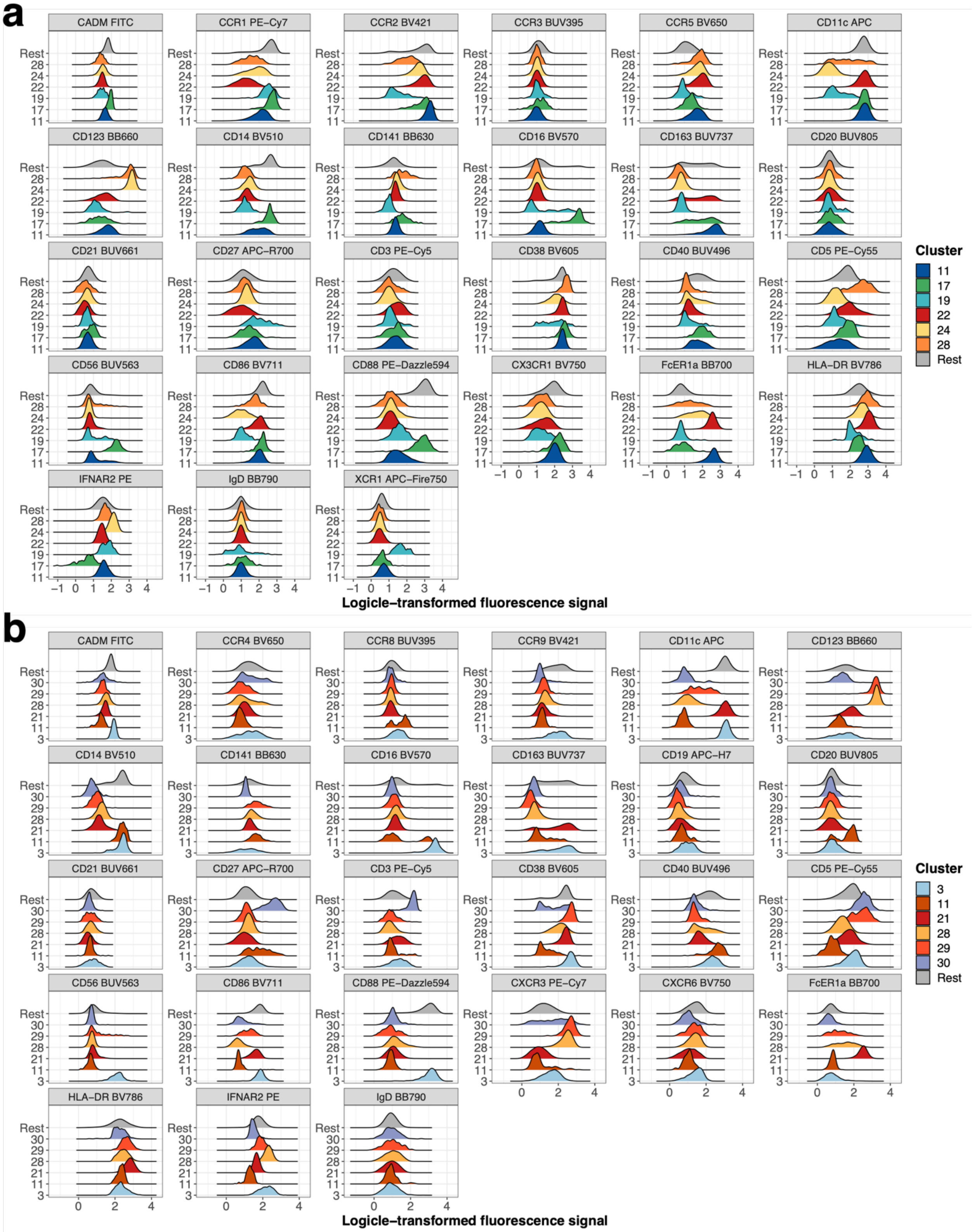
a
CADM FITC
CCR1 PE−Cy7
CCR2 BV421
CCR3 BUV395
CCR5 BV650
CD11c APC
CD123 BB660
CD14 BV510
CD141 BB630
CD16 BV570
CD163 BUV737
CD20 BUV805
CD21 BUV661
CD27 APC−R700
CD3 PE−Cy5
CD38 BV605
CD40 BUV496
CD5 PE−Cy55
CD56 BUV563
CD86 BV711
CD88 PE−Dazzle594
CX3CR1 BV750
FcER1a BB700
HLA−DR BV786
IFNAR2 PE
IgD BB790
XCR1 APC−Fire750
Rest
28
24
22
19
17
11
Cluster
11
17
19
22
24
28
Rest
−1 0 1 2 3 4
Logicle−transformed fluorescence signal
b
CADM FITC
CCR4 BV650
CCR8 BUV395
CCR9 BV421
CD11c APC
CD123 BB660
CD14 BV510
CD141 BB630
CD16 BV570
CD163 BUV737
CD19 APC−H7
CD20 BUV805
CD21 BUV661
CD27 APC−R700
CD3 PE−Cy5
CD38 BV605
CD40 BUV496
CD5 PE−Cy55
CD56 BUV563
CD86 BV711
CD88 PE−Dazzle594
CXCR3 PE−Cy7
CXCR6 BV750
FcER1a BB700
HLA−DR BV786
IFNAR2 PE
IgD BB790
Rest
30
29
28
21
11
3
Cluster
3
11
21
28
29
30
Rest
0 2 4
Logicle−transformed fluorescence signal

Supplementary Figure 15: Expression pattern of significant myeloid cell clusters between non-severe and severe COVID-19

Expression (logicle-transformed fluorescence signal) of markers from **a)** CR1 and **b)** CR2 panel for myeloid cell clusters are shown as overlaid histograms. Myeloid cell clusters are described in Figure 6 and are significantly different between individuals recovered from non-severe and severe COVID-19. All remaining clusters within myeloid cells are depicted in grey and labeled as “Rest” as a reference population.

Supplementary Table 1: Demographics summary

	Unexposed Healthy	Mild	Moderate	Severe (no ventilation)	Critical (ventilation)
Samples (N)	173	19	24	25	30
Age median (IQR)	50 (36-60)	39 (33.5-49)	50.5 (32-59)	64 (51-69)	64.5 (57.5-72.75)
Age range	18-70	26-59	22-77	37-86	43-86
Gender (F/M)	76 / 97	7 / 12	13 / 11	9 / 16	11 / 18*
Median days between symptom onset and sample collection (IQR)	NA	40 (34-57)	45.5 (34.75-78.25)	77 (34-94)	33 (25.5-48.5)
Range days between symptom onset and sample collection	NA	28-78	24-129	16-184	15-113
Hospitalized (N)	NA	1	5	23	30
Median days hospitalized (IQR)	NA	2	5 (4-6)	7 (3.5-23.5)	28 (21-46)
Range days hospitalized	NA	2	2-8	1-32	5-125
ICU (N)	NA	NA	NA	8	30

* Gender information not available for one individual.

Supplementary Table 2: Staining Panels

F/S column refers to functional (F) or subset-defining (S) marker. Marker was used for both in case of F/S.

TNK backbone			
Channel	Marker	Fluorochrome	F/S
B515	TCR Vd1	FITC	S
B610	CD127	BB630	S
B660	PD-1	BB660	F
B710	CD16	BB700	S
B780	CXCR5	BB790	S
G575	TCR Vg9	PE	S
G610	TCR Vd2	PE-CF594	S
G660	CD161	PE-Cy5	S
G710	HLA-DR	PE-Cy5.5	F/S
G780			
R670	CD1d:PBS57 tetramer	APC	S
R730	CD45RA	Ax700	S
R780			
U395			
U450	Violet L/D	UV Blue	
U500	CCR7	BUV496	S
U570	CD56	BUV563	S
U670	CD39	BUV661	F
U740	CD95	BUV737	S
U785	CD4	BUV805	S
V450			
V510	CD3	BV510	S
V570	CD8a	BV570	S
V605	CD38	BV605	F/S
V655			
V710	TCR Va7.2	BV711	S
V750			
V785	CD27	BV786	S

BDC backbone			
Channel	Marker	Fluorochrome	F/S
B515	CADM1	FITC	S
B610	CD141	BB630	S
B660	CD123	BB660	S
B710	FcEr1a	BB700	S
B780	IgD	BB790	S
G575	IFNAR2	PE	F
G610	CD88	PE-Dazzle594	S
G660	CD3	PE-Cy5	S
G710	CD5	PE-Cy5.5	S
G780			
R670	CD11c	APC	S
R730	CD27	APC-R700	S
R780			
U395			
U450	Violet L/D	UV Blue	
U500	CD40	BUV496	F
U570	CD56	BUV563	S
U670	CD21	BUV661	S
U740	CD163	BUV737	S
U785	CD20	BUV805	S
V450			
V510	CD14	BV510	S
V570	CD16	BV570	S
V605	CD38	BV605	S
V655			
V710	CD86	BV711	F
V750			
V785	HLA-DR	BV786	S

Chemokine receptor 1			
Channel	Marker	Fluorochrome	F/S
V450	CCR2	BV421	F
U395	CCR3	BUV395	F
V655	CCR5	BV650	F
V750	CX3CR1	BV750	F
G780	CCR1	PE-Cy7	F
R780	XCR1	APC-Fire750	F

Chemokine receptor 2			
Channel	Marker	Fluorochrome	F/S
V450	CCR9	BV421	F
U395	CCR8	BUV395	F
V655	CCR4	BV650	F
V750	CXCR6	BV750	F
G780	CXCR3	PE-Cy7	F
R780			

Chemokine receptor 2: BDC			
Channel	Marker	Fluorochrome	F/S
R780	CD19	APC-H7	F

Chemokine receptor 2: TNK			
Channel	Marker	Fluorochrome	F/S
R780	TIGIT	APC-Fire750	F

Supplementary Table 3: Staining reagents

Fluor-escence channel	Marker	Fluorochrome	Clone	Cat#	Lot#	Manufacturer	Amount for 50ul	Panel
U450	Viability Violet	UV Blue	-	L34962		Thermo Fisher Scientific	0.0641	All samples
B515	TCR Vd1	FITC	TS8.2	TCR2730	TA259706	Thermo Fisher Scientific	2.5	TNK backbone
B610	CD127	BB630	HIL-7R-M21	624294	1028625	BD Biosciences	1.25	TNK backbone
B660	PD-1	BB660	EH12.1	624295	1028640	BD Biosciences	0.31	TNK backbone
B710	CD16	BB700	3G8	746199	8234672	BD Biosciences (OptiBuild)	0.04	TNK backbone
B780	CXCR5	BB790	RF8B2	624296	8241555	BD Biosciences	0.04	TNK backbone
G575	TCR Vg9	PE	B3	555733	0188066	BD Biosciences	1.25	TNK backbone
G610	TCR Vd2	PE-CF594	B6	624352	8241641	BD Biosciences	0.01	TNK backbone
G660	CD161	PE-Cy5	DX12	551138	8180780	BD Biosciences	2.5	TNK backbone
G710	HLA-DR	PE-Cy5.5	TU36	MHLDR18	1990699A	Thermo Fisher Scientific	0.31	TNK backbone
R670	CD1d:PBS57 tetramer	APC	-	41386	27271P	NIH tetramer core	0.15	TNK backbone
R730	CD45RA	Ax700	HI100	560673	8164502	BD Biosciences	0.63	TNK backbone
U500	CCR7	BUV496	2-L1-A	749827	Several pre-mixed	BD Biosciences	5	TNK backbone
U570	CD56	BUV563	NCAM16.2	565704	8065832	BD Biosciences	0.31	TNK backbone
U670	CD39	BUV661	TU66	749967	Several pre-mixed	BD Biosciences	0.63	TNK backbone
U740	CD95	BUV737	DX27	624286	8242545	BD Biosciences	1.25	TNK backbone
U785	CD4	BUV805	SK3	564910	7270541	BD Biosciences	0.63	TNK backbone
V510	CD3	BV510	UCHT1	563109	8174765	BD Biosciences	0.15	TNK backbone
V570	CD8a	BV570	RPA-T8	301038	B262531	Biolegend	0.267	TNK backbone
V605	CD38	BV605	HIT2	740401	Several pre-mixed	BD Biosciences	1.25	TNK backbone
V710	TCR Va7.2	BV711	3C10	351732	B230086	Biolegend	1.25	TNK backbone
V785	CD27	BV786	L128	624292	8242923	BD Biosciences	0.31	TNK backbone
B515	CADM1	FITC	30	CM004-4	004	MBL International Corporation	0.15	BDC backbone
B610	CD141	BB630	1A4	624294	0345830	BD Biosciences	0.01	BDC backbone
B660	CD123	BB660	7G3	624295	0345827	BD Biosciences	0.04	BDC backbone
B710	FcEr1a	BB700	AER-37	747780	Several pre-mixed	BD Biosciences	0.63	BDC backbone
B780	IgD	BB790	IA6-2	624296	8241553	BD Biosciences	0.31	BDC backbone

G575	IFNAR2	PE	REA124	130-099-555	1320121071	Miltenyi	1.25	BDC backbone
G610	CD88	PE-Dazzle594	S5/1	344318	B318054	Biolegend	0.31	BDC backbone
G660	CD3	PE-Cy5	UCHT1	555334	0037612	BD Biosciences	0.31	BDC backbone
G710	CD5	PE-Cy5.5	CD5-5D7	MHCD0518	2164661	Thermo Fisher	0.31	BDC backbone
R670	CD11c	APC	B-ly6	559877	0183298	BD Biosciences	5	BDC backbone
R730	CD27	APC-R700	M-T271	624348	8242909	BD Biosciences	0.63	BDC backbone
U500	CD40	BUV496	5C3	741159	Several pre-mixed	BD Biosciences	1.25	BDC backbone
U570	CD56	BUV563	NCAM16.2	565704	8065832	BD Biosciences	0.31	BDC backbone
U670	CD21	BUV661	B-ly4	741605	Several pre-mixed	BD Biosciences	0.31	BDC backbone
U740	CD163	BUV737	GHI/61	741863	Several pre-mixed	BD Biosciences	5	BDC backbone
U785	CD20	BUV805	2H7	612905	0290068	BD Biosciences	2.5	BDC backbone
V510	CD14	BV510	MPhiP9	624289	8241676	BD Biosciences	0.1	BDC backbone
V570	CD16	BV570	3G8	302036	B314477	Biolegend	1.25	BDC backbone
V605	CD38	BV605	HIT2	740401	Several pre-mixed	BD Biosciences	1.25	BDC backbone
V710	CD86	BV711	2331	563158	0246002	BD Biosciences	0.63	BDC backbone
V785	HLA-DR	BV786	G46-6	564041	0336621	BD Biosciences	0.15	BDC backbone
V450	CCR2	BV421	48607	564067	280237	BD Biosciences	5	Chemokine receptor 1 (CR1)
U395	CCR3	BUV395	5E8	743063	Several pre-mixed	BD Biosciences	2.5	Chemokine receptor 1 (CR1)
V655	CCR5	BV650	2D7/CCR5	740600	Several pre-mixed	BD Biosciences	2.5	Chemokine receptor 1 (CR1)
V750	CX3CR1	BV750	2A9-1	747376	Several pre-mixed	BD Biosciences	5	Chemokine receptor 1 (CR1)
G780	CCR1	PE-Cy7	5F10B29	362914	B303921	Biolegend	5	Chemokine receptor 1 (CR1)
R780	XCR1	APC-Fire750	S15046E	372608	B330906	Biolegend	5	Chemokine receptor 1 (CR1)
V450	CCR9	BV421	L053E8	358914	B317814	Biolegend	5	Chemokine receptor 2 (CR2)
U395	CCR8	BUV395	433H	747573	Several pre-mixed	BD Biosciences	1.25	Chemokine receptor 2 (CR2)
V655	CCR4	BV650	1G1	744140	Several pre-mixed	BD Biosciences	5	Chemokine receptor 2 (CR2)
V750	CXCR6	BV750	13B 1E5	747052	Several pre-mixed	BD Biosciences	5	Chemokine receptor 2 (CR2)
G780	CXCR3	PE-Cy7	G025H7	353720	B300691	Biolegend	1.25	Chemokine receptor 2 (CR2)
R780	CD19	APC-H7	SC25C1	560177	0126182	BD Biosciences	0.31	Chemokine receptor 2 (CR2) - BDC
R780	TIGIT	APC-Cy7	A15153G	372734	B329043	Biolegend	0.63	Chemokine receptor 2 (CR2) - TNK

Supplementary Table 4: Markers excluded for FlowSOM analysis per immune cell lineage.

T cell / NK cell backbone (TNK)				
Panel	TNK-CR1 and TNK-CR2	TNK-CR1 and TNK-CR2	TNK-CR1 and TNK-CR2	TNK-CR1 and TNK-CR2
Lineage	CD4 T cells	CD8 T cells	Innate T cells	NK cells
Marker	Viability	Viability	Viability	Viability
	CD1d:PBS57 tetramer	CD1d:PBS57 tetramer	CD3	CD1d:PBS57 tetramer
	CD3	CD3		CD3
	CD4	CD4		TCR Vδ1
	CD8a	CD8a		TCR Vδ2
	TCR Vδ1	TCR Vδ1		TCR Vγ9
	TCR Vδ2	TCR Vδ2		TCR Vα7.2
	TCR Vγ9	TCR Vγ9		HLA-DR

B cell / myeloid cell backbone (BDC)				
Panel	BDC-CR1	BDC-CR2	BDC-CR1	BDC-CR2
Lineage	Myeloid cells	Myeloid cells	B cells	B cells
Marker	Viability	Viability	Viability	Viability
	CD3	CD3	CADM1	CADM1
	CD20	CD19	CD141	CD141
	CD21	CD20	CD123	CD123
	IgD	CD21	FcεR1a	FcεR1a
		IgD	CD88	CD88
			CD3	CD3
			CD56	CD19
			CD163	CD56
			CD14	CD163
			CD16	CD14
			HLA-DR	CD16
				HLA-DR

Supplementary Table 5: FlowSOM clusters with potential immune cell contaminants derived from other lineages removed from subsequent analysis.

Contaminating immune populations - FlowSOM clusters removed from subsequent analysis												
Panel	BDC-CR1	BDC-CR2	BDC-CR1	BDC-CR2	TNK-CR1	TNK-CR2	TNK-CR1	TNK-CR2	TNK-CR1	TNK-CR2	TNK-CR1	TNK-CR2
Lineage	B cells	B cells	Myeloid cells	Myeloid cells	CD4 T cells	CD4 T cells	CD8 T cells	CD8 T cells	Innate T cells	Innate T cells	NK cells	NK cells
Clusters	all clusters included	all clusters included	all clusters included	all clusters included	23	11	18	6	20	17	1	1
					38	22	28	34			5	2
											7	3
											9	4
											11	7
											18	8
											20	14
											22	15
											23	16
											25	17
											29	19

Supplementary Table 6: Buffers and cell culture media used in this study.

Item	Vendor	Catalog#
RPMI w/o phenol red	Thermo Fisher Scientific	A14576DK
Newborn Calf Serum, Heat Inactivated (HINCS)	R&D Systems	S11250H
Brilliant Stain Buffer Plus	BD Biosciences	566385
True-Stain Monocyte Blocker	Biolegend	426103
RPMI 1640 medium, no glutamine	Thermo Fisher Scientific	21870092
Fetal Bovine Serum, heat Inactivated (FBS)	Thermo Fisher Scientific	10438026
Penicillin-Streptomycin-Glutamine (100X)	Thermo Fisher Scientific	10378016
Benzonase	Millipore Sigma	71205-25KUN
DPBS, no calcium, no magnesium	Thermo Fisher Scientific	14190-250
Human BD Fc Block (250ug)	BD Biosciences	564220
20% Formaldehyde	Tousimis	1008B
96-well, V-bottom plates	Corning	3894
