# Supplementary material for: Immune phenotypes that are associated with subsequent COVID-19 severity inferred from post-recovery samples
Source: Nat Commun. 2022 Nov 25;13:7255. doi: 10.1038/s41467-022-34638-2 (PMC9700777; doi:10.1038/s41467-022-34638-2)
Supplement: Supplementary file 3 — Reporting Summary [file 41467_2022_34638_MOESM3_ESM.pdf]

nature portfolio

Corresponding author(s): Dr. Mario Roederer

Last updated by author(s): Sep 30, 2022

Reporting Summary

Nature Portfolio wishes to improve the reproducibility of the work that we publish. This form provides structure for consistency and transparency in reporting. For further information on Nature Portfolio policies, see our Editorial Policies and the Editorial Policy Checklist.

Statistics

For all statistical analyses, confirm that the following items are present in the figure legend, table legend, main text, or Methods section.

n/a	Confirmed	
☐	☒	The exact sample size (n) for each experimental group/condition, given as a discrete number and unit of measurement
☐	☒	A statement on whether measurements were taken from distinct samples or whether the same sample was measured repeatedly
☐	☒	The statistical test(s) used AND whether they are one- or two-sided<br>*Only common tests should be described solely by name; describe more complex techniques in the Methods section.*
☐	☒	A description of all covariates tested
☐	☒	A description of any assumptions or corrections, such as tests of normality and adjustment for multiple comparisons
☐	☒	A full description of the statistical parameters including central tendency (e.g. means) or other basic estimates (e.g. regression coefficient) AND variation (e.g. standard deviation) or associated estimates of uncertainty (e.g. confidence intervals)
☐	☒	For null hypothesis testing, the test statistic (e.g. F, t, r) with confidence intervals, effect sizes, degrees of freedom and P value noted<br>*Give P values as exact values whenever suitable.*
☒	☐	For Bayesian analysis, information on the choice of priors and Markov chain Monte Carlo settings
☐	☒	For hierarchical and complex designs, identification of the appropriate level for tests and full reporting of outcomes
☒	☐	Estimates of effect sizes (e.g. Cohen's d, Pearson's r), indicating how they were calculated

Our web collection on statistics for biologists contains articles on many of the points above.

Software and code

Policy information about availability of computer code

Data collection	Data was collected using a BD FACSymphony cytometer with FACS DIVA v9.1 acquisition software.
Data analysis	FlowJo (v10.1.7 and v10.7.2), R (v4.0.0), R packages (FlowAI, v1.18.5; missCompare, v1.0.3; Rtsne, v0.15; FlowSOM, v1.20.0)

For manuscripts utilizing custom algorithms or software that are central to the research but not yet described in published literature, software must be made available to editors and reviewers. We strongly encourage code deposition in a community repository (e.g. GitHub). See the Nature Portfolio guidelines for submitting code & software for further information.

Data

Policy information about availability of data

All manuscripts must include a data availability statement. This statement should provide the following information, where applicable:

- Accession codes, unique identifiers, or web links for publicly available datasets
- A description of any restrictions on data availability
- For clinical datasets or third party data, please ensure that the statement adheres to our policy

The raw data flow cytometry files (corrected for fluorescence spillover and pre-gated on viable cells) are available on FlowRepository: http://flowrepository.org/id/FR-FCM-Z5PC.

Field-specific reporting

Please select the one below that is the best fit for your research. If you are not sure, read the appropriate sections before making your selection.

☒ Life sciences ☐ Behavioural & social sciences ☐ Ecological, evolutionary & environmental sciences

For a reference copy of the document with all sections, see nature.com/documents/nr-reporting-summary-flat.pdf

Life sciences study design

All studies must disclose on these points even when the disclosure is negative.

Sample size	We aimed for samples with longest time period between symptom onset and sample collection. Sample size was dependent on access to such samples. 173 healthy individuals were selected based on matching distribution of gender, ethnicity and age. No sample size calculation was performed.
Data exclusions	Data from two individuals were excluded based on insufficient cell numbers. Slight signal shifts occurred for two samples and one of the staining panels (BDC-CR1). This did not affect the manual definition of immune cell population but we excluded data from those two samples and staining panel for unsupervised data analysis (FlowSOM and tSNE). Such signal shifts can be accommodated with manual gating by applying sample-specific cut-offs for positive signal (i.e. presence of expression). Sample-specific cut-off for expression signal is not possible for unsupervised analysis and therefore signal shifts will result in artificial definition of clusters by the clustering algorithms.
Replication	Samples were measured in two batches. PBMCs from the same donor and batch of cells (QC sample) were included in both batches. Reproducibility was assessed by inspecting the difference in immune traits (Frequency and MFI values) between these QC samples from the two batches. We did not observe substantial differences between the QC sample from the two experiments. This was also reflected by the fact that we did not observe large differences in signal intensities between the two experiments during analysis.
Randomization	Samples from each COVID-19 severity group and healthy individuals were equally distributed across the two experiments to minimize batch effects. Samples were measured within two experiments and samples from study groups (unexposed healthy controls and individuals recovered from mild, moderate, severe and critical) were evenly distributed across the two experiments to avoid measurement bias between study groups.
Blinding	Blinding was not performed in this study since samples from individuals recovered from COVID-19 with various degrees of disease severity were rare and difficult to obtain.

Reporting for specific materials, systems and methods

We require information from authors about some types of materials, experimental systems and methods used in many studies. Here, indicate whether each material, system or method listed is relevant to your study. If you are not sure if a list item applies to your research, read the appropriate section before selecting a response.

Materials & experimental systems

n/a	Involved in the study	
☐	☒	Antibodies
☒	☐	Eukaryotic cell lines
☒	☐	Palaeontology and archaeology
☒	☐	Animals and other organisms
☐	☒	Human research participants
☒	☐	Clinical data
☒	☐	Dual use research of concern

Methods

n/a	Involved in the study	
☒	☐	ChIP-seq
☐	☒	Flow cytometry
☒	☐	MRI-based neuroimaging

Antibodies

Antibodies used	Staining reagents with information about manufacturer, catalog number, lot number, concentration used and clone are listed in supplementary table 2. Staining reagents included:<br><br>Viability UV Blue (Manufacturer: Thermo Fisher Scientific, Cat#: L34962, titer per 50ul staining volume: 0.0641ul), anti-TCR Vd1 FITC (clone: TS8.2, Manufacturer: Thermo Fisher Scientific, Cat#: TCR2730, titer per 50ul staining volume: 2.5ul), anti-CD127 BB630 (clone: HIL-7R-M21, Manufacturer: BD Biosciences, Cat#: 624294, titer per 50ul staining volume: 1.25ul), anti-PD-1 BB660 (clone: EH12.1, Manufacturer: BD Biosciences, Cat#: 624295, titer per 50ul staining volume: 0.31ul), anti-CD16 BB700 (clone: 3G8, Manufacturer: BD Biosciences (OptiBuild), Cat#: 746199, titer per 50ul staining volume: 0.04ul), anti-CXCR5 BB790 (clone: RF8B2, Manufacturer: BD Biosciences, Cat#: 624296, titer per 50ul staining volume: 0.04ul), anti-TCR Vg9 PE (clone: B3, Manufacturer: BD Biosciences, Cat#: 555733, titer per 50ul staining volume: 1.25ul), anti-TCR Vd2 PE-CF594 (clone: B6, Manufacturer: BD Biosciences, Cat#: 624352, titer per 50ul staining volume: 0.01ul), anti-CD161 PE-Cy5 (clone: DX12, Manufacturer: BD Biosciences, Cat#: 551138, titer per 50ul staining volume: 2.5ul), anti-HLA-DR PE-Cy5.5 (clone: TU36, Manufacturer: Thermo Fisher Scientific, Cat#: MHLDR18, titer per 50ul

staining volume: 0.31ul), anti-CD1d:PBS57 tetramer APC (clone: -, Manufacturer: NIH tetramer core, Cat#: 41386, titer per 50ul staining volume: 0.15ul), anti-CD45RA Ax700 (clone: HI100, Manufacturer: BD Biosciences, Cat#: 560673, titer per 50ul staining volume: 0.63ul), anti-CCR7 BUV496 (clone: 2-L1-A, Manufacturer: BD Biosciences, Cat#: 749827, titer per 50ul staining volume: 5ul), anti-CD56 BUV563 (clone: NCAM16.2, Manufacturer: BD Biosciences, Cat#: 565704, titer per 50ul staining volume: 0.31ul), anti-CD39 BUV661 (clone: TU66, Manufacturer: BD Biosciences, Cat#: 749967, titer per 50ul staining volume: 0.63ul), anti-CD95 BUV737 (clone: DX27, Manufacturer: BD Biosciences, Cat#: 624286, titer per 50ul staining volume: 1.25ul), anti-CD4 BUV805 (clone: SK3, Manufacturer: BD Biosciences, Cat#: 564910, titer per 50ul staining volume: 0.63ul), anti-CD3 BV510 (clone: UCHT1, Manufacturer: BD Biosciences, Cat#: 563109, titer per 50ul staining volume: 0.15ul), anti-CD8a BV570 (clone: RPA-T8, Manufacturer: Biolegend, Cat#: 301038, titer per 50ul staining volume: 0.267ul), anti-CD38 BV605 (clone: HIT2, Manufacturer: BD Biosciences, Cat#: 740401, titer per 50ul staining volume: 1.25ul), anti-TCR Va7.2 BV711 (clone: 3C10, Manufacturer: Biolegend, Cat#: 351732, titer per 50ul staining volume: 1.25ul), anti-CD27 BV786 (clone: L128, Manufacturer: BD Biosciences, Cat#: 624292, titer per 50ul staining volume: 0.31ul), anti-CADM1 FITC (clone: 30, Manufacturer: MBL International Corporation, Cat#: CM004-4, titer per 50ul staining volume: 0.15ul), anti-CD141 BB630 (clone: 1A4, Manufacturer: BD Biosciences, Cat#: 624294, titer per 50ul staining volume: 0.01ul), anti-CD123 BB660 (clone: 7G3, Manufacturer: BD Biosciences, Cat#: 624295, titer per 50ul staining volume: 0.04ul), anti-FcEr1a BB700 (clone: AER-37, Manufacturer: BD Biosciences, Cat#: 747780, titer per 50ul staining volume: 0.63ul), anti-IgD BB790 (clone: IA6-2, Manufacturer: BD Biosciences, Cat#: 624296, titer per 50ul staining volume: 0.31ul), anti-IFNAR2 PE (clone: REA124, Manufacturer: Miltenyi, Cat#: 130-099-555, titer per 50ul staining volume: 1.25ul), anti-CD88 PE-Dazzle594 (clone: S5/1, Manufacturer: Biolegend, Cat#: 344318, titer per 50ul staining volume: 0.31ul), anti-CD3 PE-Cy5 (clone: UCHT1, Manufacturer: BD Biosciences, Cat#: 555334, titer per 50ul staining volume: 0.31ul), anti-CD5 PE-Cy5.5 (clone: CD5-5D7, Manufacturer: Thermo Fisher, Cat#: MHCD0518, titer per 50ul staining volume: 0.31ul), anti-CD11c APC (clone: B-ly6, Manufacturer: BD Biosciences, Cat#: 559877, titer per 50ul staining volume: 5ul), anti-CD27 APC-R700 (clone: M-T271, Manufacturer: BD Biosciences, Cat#: 624348, titer per 50ul staining volume: 0.63ul), anti-CD40 BUV496 (clone: 5C3, Manufacturer: BD Biosciences, Cat#: 741159, titer per 50ul staining volume: 1.25ul), anti-CD56 BUV563 (clone: NCAM16.2, Manufacturer: BD Biosciences, Cat#: 565704, titer per 50ul staining volume: 0.31ul), anti-CD21 BUV661 (clone: B-ly4, Manufacturer: BD Biosciences, Cat#: 741605, titer per 50ul staining volume: 0.31ul), anti-CD163 BUV737 (clone: GHI/61, Manufacturer: BD Biosciences, Cat#: 741863, titer per 50ul staining volume: 5ul), anti-CD20 BUV805 (clone: 2H7, Manufacturer: BD Biosciences, Cat#: 612905, titer per 50ul staining volume: 2.5ul), anti-CD14 BV510 (clone: MPhiP9, Manufacturer: BD Biosciences, Cat#: 624289, titer per 50ul staining volume: 0.1ul), anti-CD16 BV570 (clone: 3G8, Manufacturer: Biolegend, Cat#: 302036, titer per 50ul staining volume: 1.25ul), anti-CD38 BV605 (clone: HIT2, Manufacturer: BD Biosciences, Cat#: 740401, titer per 50ul staining volume: 1.25ul), anti-CD86 BV711 (clone: 2331, Manufacturer: BD Biosciences, Cat#: 563158, titer per 50ul staining volume: 0.63ul), anti-HLA-DR BV786 (clone: G46-6, Manufacturer: BD Biosciences, Cat#: 564041, titer per 50ul staining volume: 0.15ul), anti-CCR2 BV421 (clone: 48607, Manufacturer: BD Biosciences, Cat#: 564067, titer per 50ul staining volume: 5ul), anti-CCR3 BUV395 (clone: 5E8, Manufacturer: BD Biosciences, Cat#: 743063, titer per 50ul staining volume: 2.5ul), anti-CCR5 BV650 (clone: 2D7/CCR5, Manufacturer: BD Biosciences, Cat#: 740600, titer per 50ul staining volume: 2.5ul), anti-CX3CR1 BV750 (clone: 2A9-1, Manufacturer: BD Biosciences, Cat#: 747376, titer per 50ul staining volume: 5ul), anti-CCR1 PE-Cy7 (clone: 5F10B29, Manufacturer: Biolegend, Cat#: 362914, titer per 50ul staining volume: 5ul), anti-XCR1 APC-Fire750 (clone: S15046E, Manufacturer: Biolegend, Cat#: 372608, titer per 50ul staining volume: 5ul), anti-CCR9 BV421 (clone: L053E8, Manufacturer: Biolegend, Cat#: 358914, titer per 50ul staining volume: 5ul), anti-CCR8 BUV395 (clone: 433H, Manufacturer: BD Biosciences, Cat#: 747573, titer per 50ul staining volume: 1.25ul), anti-CCR4 BV650 (clone: 1G1, Manufacturer: BD Biosciences, Cat#: 744140, titer per 50ul staining volume: 5ul), anti-CXCR6 BV750 (clone: 13B 1E5, Manufacturer: BD Biosciences, Cat#: 747052, titer per 50ul staining volume: 5ul), anti-CXCR3 PE-Cy7 (clone: G025H7, Manufacturer: Biolegend, Cat#: 353720, titer per 50ul staining volume: 1.25ul), anti-CD19 APC-H7 (clone: SC25C1, Manufacturer: BD Biosciences, Cat#: 560177, titer per 50ul staining volume: 0.31ul), anti-TIGIT APC-Cy7 (clone: A15153G, Manufacturer: Biolegend, Cat#: 372734, titer per 50ul staining volume: 0.63ul)

Validation

We extensively tested these staining panels for optimal resolution and biologically meaningful staining pattern. These panels are part of previously published and in detail described staining panels (OMIP-051, 10.1002/cyto.a.23689; OMIP-058, 10.1002/cyto.a.23850).

Human research participants

Policy information about studies involving human research participants

Population characteristics

Demographic information is specified in Extended Data Figure 2.

Recruitment

Healthy donors were recruited as part of the Vaccine Research clinical program. Samples from individuals recovered from mild and moderate COVID-19 were collected during treatment follow-up either at NIH or the Evergreen clinic, WA. Samples from severe and critical COVID-19 cases were taken during treatment follow-up at the hospital.

Ethics oversight

IRB of NIH VRC and Washington University

Note that full information on the approval of the study protocol must also be provided in the manuscript.

Flow Cytometry

Plots

Confirm that:

- [x] The axis labels state the marker and fluorochrome used (e.g. CD4-FITC).
- [x] The axis scales are clearly visible. Include numbers along axes only for bottom left plot of group (a 'group' is an analysis of identical markers).
- [x] All plots are contour plots with outliers or pseudocolor plots.
- [x] A numerical value for number of cells or percentage (with statistics) is provided.

Methodology

Sample preparation	Sample processing is outlined in detail in the method section. Briefly, samples were thawed using the thawsome tube adaptor into cell culture medium (RPMI1640, 10% FCS and Penicillin/Streptomycin). Subsequently, samples were stained with Live/Dead UV Blue viability dye containing Human BD Fc Block (BD Biosciences, Cat# 564220) and TrueStain Monocyte Blocker (BioLegend, Cat# 426103) reagent for 20 minutes at room temperature. Afterwards, cells were stained for 30 minutes with antibody cocktail containing 1:5 diluted Brilliant Stain Buffer Plus (BD Biosciences, Cat# 566385) before cells were fixed with 0.5% paraformaldehyde and acquired with a BD FACSymphony flow cytometer.
Instrument	Flow cytometry data was collected with a BD FACSymphony cytometer. Configuration and specifications can be found elsewhere in detail (10.1002/cyto.a.23689).
Software	Samples were acquired with FACS Diva version 9.1.
Cell population abundance	No sorting was performed for this study.
Gating strategy	Cells were defined based on Forward (FSC-A) and Side Scatter (SSC-A) parameters and cell aggregates were excluded using Forward scatter height (FSC-H) versus area (FSC-A) signal. Subsequently, viable cells were further defined by the absence of staining with viability dye. All other subsequent gating strategies to define immune cell subsets are visualized in the supplementary part.

[x] Tick this box to confirm that a figure exemplifying the gating strategy is provided in the Supplementary Information.
